# Digital mental health apps for young people in Europe: A systematic cross-country analysis

**DOI:** 10.1371/journal.pdig.0001622

**Published:** 2026-08-27

**Authors:** Alba Madrid Cagigal, Canan Citil Akyol, Nesime Can Angın, João Ferreira, Karolina Gabor-Siatkowska, Niko Männikkö, Anna Meyniel, Neslihan Önder Özdemir, Miloš Stojadinović, Vanesa Varga, Ine Beyens, Claudette Pretorius

**Affiliations:** 1 School of Psychology, University of Galway, Galway, Ireland; 2 Department of Psychology, Faculty of Letters, Sivas Cumhuriyet University, Sivas, Türkiye; 3 Program of Guidance and Counseling, Faculty of Educational Sciences, Ankara University, Ankara, Türkiye; 4 Institute of Pharmacology and Experimental Therapeutics, Coimbra Institute for Clinical and Biomedical Research (iCBR), Faculty of Medicine, University Coimbra, Coimbra, Portugal; 5 Center for Innovative Biomedicine and Biotechnology (CIBB), Univ Coimbra, Coimbra, Portugal; 6 Institute of Telecommunications and Cybersecurity, Faculty of Electronics and Information Technology, Warsaw University of Technology, Warsaw, Poland; 7 Centre for Research and Innovation; Oulu University of Applied Sciences, Oulu, Finland; 8 International Centre for Missing and Exploited Children, Alexandria, Virginia, United States of America; 9 School of Foreign Languages, Bursa Uludağ University, Bursa, Türkiye; 10 Department of Psychology, Faculty of Philosophy, University of Niš, Niš, Serbia; 11 Faculty of Croatian Studies, University of Zagreb, Department for Communication Studies, Zagreb, Croatia; 12 Amsterdam School of Communication Research, University of Amsterdam, Amsterdam, The Netherlands; 13 School of Computer Science, University College Dublin, Dublin, Ireland; Wollo University, ETHIOPIA

## Abstract

Mental health smartphone applications have become widely available for self-management and support, offering young people new pathways for help-seeking. Despite their increasing popularity, little is known about how app accessibility, content, and features vary across countries, languages, and cultural contexts. This study aimed to systematically examine these differences by drawing on data from the available app stores. Both the Apple App Store and Google Play Store were searched across seven European countries (Croatia, France, Ireland, Poland, Portugal, Serbia, and Türkiye) using four keywords: mental health, psychological wellbeing, self-help, and stress. App results were screened through redefined inclusion and exclusion criteria, and the included apps were systematically characterized based on their aims, features, and linguistic accessibility. Over 900 apps were initially identified, and 171 met the inclusion criteria. The most commonly encountered apps were structured journaling and meditation apps, and most were partially paid and primarily available in English, highlighting a predominance of commercially oriented apps and a limited availability of local-language apps and minimal cultural adaptation. Together, the findings underscore the need for research and policy to prioritize cross-country accessibility, particularly for non-English speaking youth, to ensure equitable access to digital mental health resources.

## Author summary

Digital mental health interventions have rapidly expanded, offering young people new ways to access mental health support and manage their wellbeing. However, little is known about how online mental health-related applications differ across languages, cultures and countries. To address this gap, in this study, we systematically searched the Apple App Store and Google Play Store in seven European countries (Croatia, France, Ireland, Portugal, Serbia and Türkiye) using both English and local-language keywords related to mental health and wellbeing. We identified 171 mental health-related applications and analyzed their aims, features, and linguistic accessibility. Our findings revealed that commercial English-language apps featuring mindfulness and journaling tools were the most common, with limited cultural or linguistic adaptations and a scarcity of locally developed apps. The results further suggest that young people searching for mental health-related applications in their native language are most likely to encounter apps aimed at managing stress or enhancing wellbeing, rather than apps that address significant mental health difficulties. Together, the findings underscore the need to promote equitable access to culturally relevant and evidence-based digital resources, ensuring that digital mental health interventions effectively support young people across diverse European contexts.

## Introduction

The World Health Organization [1] defines mental health as a state characterized by the presence of wellbeing alongside the absence of ill-being. It has highlighted the role digital interventions can play in complementing and expanding mental health care systems. The field of digital mental health has rapidly expanded, encompassing smartphone applications (apps), web-based programs, virtual reality environments, chatbots and artificial intelligence (AI), including large language models (LLMs). Among these digital tools, smartphone apps have experienced a rapid adoption, serving as an accessible tool that individuals can use to self-manage or complement mental health care [2]. Some apps even function as medical devices, supporting digital phenotyping and real-time data collection through ecological momentary assessments and sensor-derived behavioral metrics [3]. However, due to the rapid expansion of the app marketplace, currently hosting over 10,000 mental health-related apps, research examining their aims, features, and linguistic accessibility has lagged [4].

Mental health apps commonly include features such as mindfulness exercises, meditation, journaling and mood-tracking [5,6]. Commercially popular apps, such as Calm, Headspace, and Wysa, are widely used and often provide short-term symptom relief [7]. Given their widespread availability, mental health applications are an essential tool for help-seeking, the process of actively seeking support and information from both informal and formal sources when experiencing psychological distress [8]. Offering functions, such as psychoeducation, symptom monitoring, and guided self-help, mental health apps provide new pathways for help-seeking among young people who might not otherwise engage with traditional mental health services. In fact, despite the established benefits of help-seeking, many young people remain reluctant to access offline mental health support [9]. Reported barriers include perceived stigma, concerns about confidentiality, lack of accessibility and affordability, and preference for self-reliance and anonymity [10]. Mobile applications can address these barriers, offering accessible, user-controlled, and non-judgemental environments that align with young people’s preferences for privacy and self-reliance [11].

Despite these potential benefits that mental health applications may bring, several critical gaps persist in the literature. Most notably, little is known about cross-country and cross-language variations in app availability and presentation. Prior research has highlighted persistent usability and engagement challenges of mental health-related apps [12–14]. Many studies have investigated these challenges in apps targeting a specific mental health condition such as depression [15], schizophrenia [16], and anxiety [17]. However, few have examined how language, culture, and regional context influence young people’s access to mental health apps across international settings [18,19]. Previous research has shown that apps highly visible in English-language stores may be less accessible or miscategorized in local-language stores, resulting in digital access inequalities [20]. These gaps underscore the need for research to evaluate linguistic and cultural accessibility across countries.

The present study addresses these gaps by conducting a cross-country and cross-language comparison of mental health apps accessible to young people across seven European countries: Croatia, France, Ireland, Poland, Portugal, Serbia, and Türkiye. Specifically, this study examines differences in app availability, features, and presentation between English-language and local-language searches, evaluating cultural and linguistic accessibility, and investigating how keyword selection and app categorization vary across app stores and regions. This study also provides a closer examination of the types of apps available and the specific features they offer, enabling a more detailed understanding of how mental health support is delivered across various digital marketplaces. By focusing on these dimensions, this research strengthens the evidence base for equitable digital mental health access, informs culturally sensitive app development and implementation policy, and situates smartphone-based interventions within broader help-seeking frameworks for young people.

## Methods

### Search strategy

To map the availability of mental health–related apps, we conducted a systematic search of the iOS (Apple Store) and Android (Google Play) platforms, reporting results using the PRISMA guidelines. Similar methodologies have been used in prior studies. [21,22]. Apps were searched in the Apple App Store and Google Play Store between April 28 and May 5, 2025. We developed a list of predefined self-help-related keywords informed by prior literature [5,12,23]. After discussion within the research team, four final keywords were selected (“mental health,” “psychological wellbeing,” “self-help,” and “stress”) to reflect search terms that young people are most likely to use when seeking digital mental health support.

Searches were conducted by authors with expertise in digital mental health and youth mental health across seven countries: Croatia, France, Ireland, Poland, Portugal, Serbia, and Türkiye.This study focuses on seven European countries (Croatia, France, Ireland, Poland, Portugal, Serbia, and Türkiye) selected through purposive sampling to capture heterogeneity rather than to provide exhaustive coverage of all European countries. The countries were chosen to reflect diversity in geographic regions (Western, Southern, and Central/Eastern Europe), political and health system contexts, and linguistic and cultural environments. We also prioritised countries where the research team had sufficient linguistic and contextual expertise to systematically search, screen, and interpret app content in the local language(s). Consequently, the seven countries should be understood as an illustrative sample of European contexts, rather than a representative selection of all European countries.

In Ireland, searches were performed only in English, the primary language of the app store. In the remaining countries, two parallel searches were conducted: one using English keywords and another employing translations into the respective national language. Conducting searches in both languages enabled the identification of apps that may be marketed differently across linguistic contexts, thereby reducing the risk of missing relevant, locally targeted apps.

Each researcher conducted searches via their respective national app-store settings. To minimise algorithmic bias, personalized recommendations and search history features were disabled prior to data collection. This approach reflected realistic user conditions while limiting the potential influence of prior browsing activity on search results. To ensure comparability and to capture the apps most likely to be encountered by users, only the first ten results returned for each search were included. This threshold likely reflects the real-life behaviour of young people [12,24,25]. The full output of the initial searches is available in S1 Table.

In this study, “young people” was defined as individuals aged 11–25. This age range captures the key developmental transitions from childhood to adolescence and from adolescence to early adulthood, during which the majority of mental health difficulties emerge: 75% of mental health conditions have their onsent before the age of 25 [26]. It is important to note that app store classifications do not typically align with this definition. Instead, apps are categorised using broad age ratings (e.g., 4 + , 12 + , 18+), which do not necessarily reflect developmental appropriateness or suitability for youth mental health support. Therefore, age-related classifications reported in this study reflect app store metadata rather than verified target populations. Importantly, this study did not assume that apps categorised as “4+” or “12+” were specifically designed for children or adolescents. Rather, these classifications were used to examine which apps are broadly accessible and visible to young people within commercial app stores. Where app descriptions explicitly identified a target population (e.g., young adults, or families), this information was extracted separately.

### Data extraction procedure

For each keyword, datasets were generated, including app results from all seven countries. A structured codebook was developed to guide the systematic extraction of data from each app. The following information was coded: app name, primary aim, key features, app store, cost model, age rating, date of last update, user rating, number of downloads, and any declared implementation of AI.

**Fig 1:**
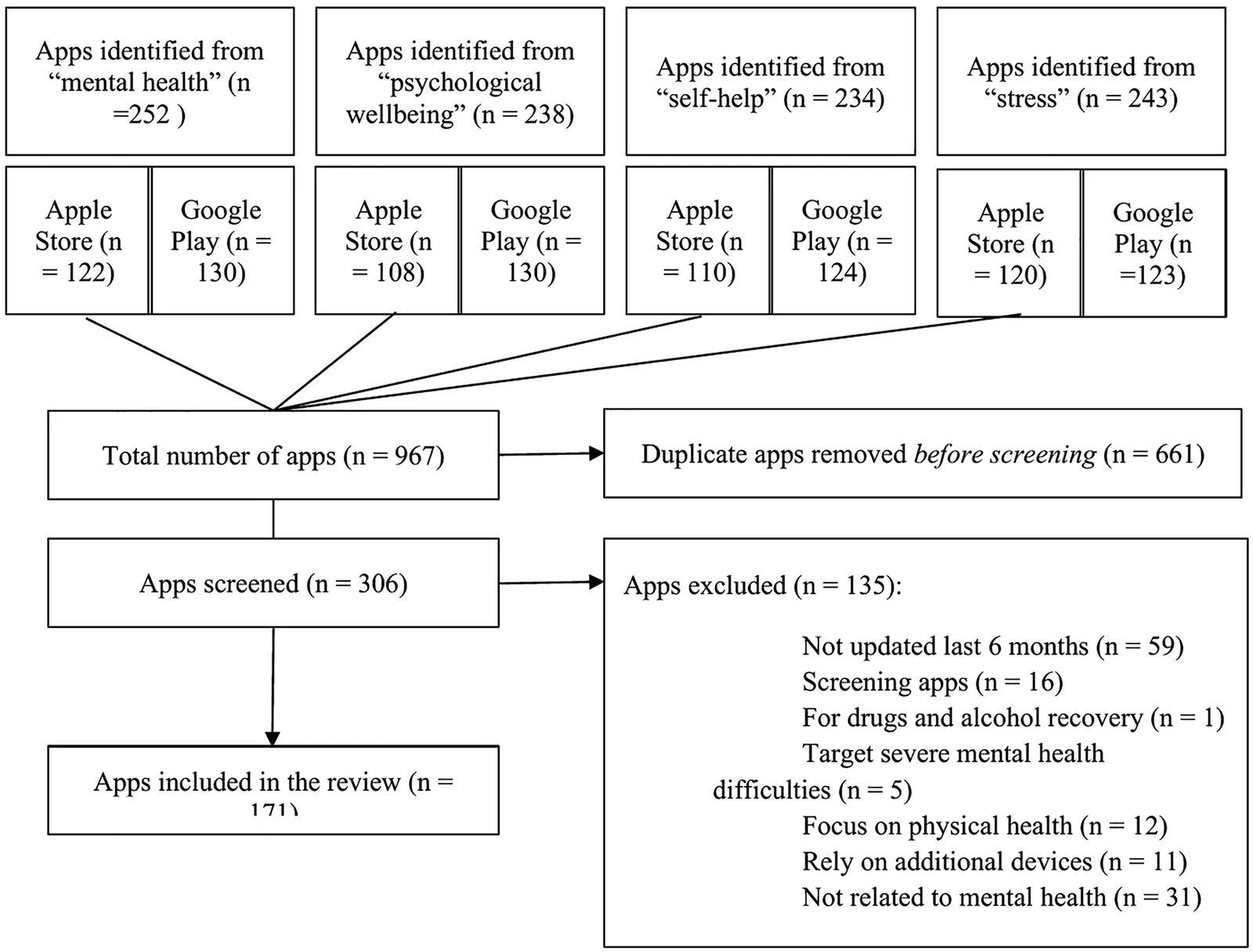
PRISMA flow diagram of apps included within the search.

As shown in the PRISMA flowchart (Fig 1), a total of 967 apps were identified across all keywords and countries during the app store searches (460 apps from Apple App Store, and 507 apps from the Google Play Store). After removing 661 duplicate entries, 306 unique apps were retained and screened. Predefined exclusion and inclusion criteria were applied for screening the apps (see Table 1). Apps with the most recent update dating back more than six months (i.e., before September 2024) were excluded to avoid including apps that were no longer actively used and/or regularly reviewed and updated. Additional apps were excluded if their primary focus was unrelated to mental health or psychological wellbeing, or if they targeted only physical health conditions. Following this screening process, 171 apps met all criteria and were retained for final analysis.

**Table 1:** Inclusion and exclusion criteria used to identify eligible mental health-related applications from the app stores.

Inclusion Criteria	Exclusion Criteria
• The store description of the app indicates that the app aims to provide support for any aspect of psychological wellbeing/mental health/mental wellbeing, and/or decreasing anxiety and/or depression and/or stress. • Last updated within the last 6 months.	• Screening apps that only include screening measures like the PHQ-9, DASS and other diagnostic tools. • Apps targeting only drug or alcohol recovery. • Apps that only target severe mental health difficulties, such as personality or eating disorders. • Apps that only focus on physical health. • Apps that rely on additional devices (e.g., wearable heart-rate monitors or watches). • Apps not updated within the last 6 months.

All included apps were compiled into a single spreadsheet for subsequent categorization and analysis. Based on information provided in the app store descriptions, each app was characterized according to its purpose, target population, cost structure, language availability, and declared AI integration. It should be noted that only the Apple App Store, and not the Google Play Store, explicitly lists supported languages.

## Results

### Cross-country app visibility

The review of the 171 apps revealed several similarities in the visibility of apps across countries and search terms. In particular, English-language and commercial apps were dominant. The same high-visibility commercial English-language applications frequently appeared in the search results across the seven countries, including Headspace, Wysa, MindDoc, VOS (a mood and wellbeing tracking app) and Daylio. This overlap indicates a strong global market presence for English-language commercial mental health apps. In addition, searches conducted in non-English languages (Croatian, French, Polish, Portuguese, Serbian and Turkish) largely returned English-language apps, with relatively few locally developed or language-specific options available. In several cases, particularly for Serbian and Croatian searches, specific keywords, such as *“self-help”* or *“psychological wellbeing,”* returned very few or no results.

Cross-country discrepancies were also observed in app store metadata. These differences suggest that app visibility and user engagement metrics may vary across national app store contexts, even for globally dominant applications, showing that even the same app is presented differently to users depending on which country’s store they access. For example, Headspace, the most globally visible app in our sample, showed notable variation in user ratings and review counts across country-specific Apple Store listings (Table 2), indicating that app “popularity” as indexed by ratings is not directly comparable across national contexts.

**Table 2:** Example of country-specific variation in Apple App Store data for Headspace.

Link including geolocation prefix	Country represented by prefix	Rating	Number of ratings
https://apps.apple.com/**hr**/app/headspace-sleep-meditation/id493145008	Croatia	4.9	1.4K
https://apps.apple.com/**fr**/app/headspace-mindfulness-sant%C3%A9/id493145008	France	4.8	21.4K
https://apps.apple.com/**ie**/app/headspace-sleep-meditation/id493145008	Ireland	4.7	24.1K
https://apps.apple.com/**pl**/app/headspace-sleep-meditation/id493145008	Poland	4.9	7.6K
https://apps.apple.com/**pt**/app/headspace-sono-e-medita%C3%A7%C3%A3o/id493145008	Portugal	4.8	8.3K
https://apps.apple.com/**rs**/app/headspace-sleep-meditation/id493145008	Serbia	4.7	216
https://apps.apple.com/**tr**/app/headspace-sleep-meditation/id493145008	Türkiye	4.8	5.4K

### Cross-language and cross-country differences

Cross-country differences were most evident in the contrast between English-language and native-language searches. English-language searches consistently returned a broader range of commercially dominant apps across all countries, including Headspace, Wysa, Daylio, MindDoc, and BetterMe. In contrast, searches conducted in local languages frequently yielded fewer results, particularly in Croatia and Serbia, where some translated keywords returned minimal or no relevant applications. For example, Serbian-language searches using terms related to “*psychological wellbeing*” returned no Apple App Store results for several keywords, whereas Croatian-language searches often returned only a small number of locally relevant applications.

Differences were also observed between countries in the extent of localisation and cultural adaptation. French- and Portuguese-language searches generally returned a broader range of locally adapted or partially localised apps, including apps available in French or Portuguese interfaces, whereas searches conducted in Serbian, Croatian, and Turkish more commonly returned English-language commercial apps with limited localisation beyond translation of the app title or description.

Native-language searches were also more likely to return apps focused on general stress reduction, self-help, or wellbeing rather than apps describing therapeutic or clinically oriented support. Across countries, relatively few apps appeared to be specifically tailored to local cultural or linguistic contexts. Most apps identified through local-language searches were either direct translations of English-language commercial apps or applications with limited localisation beyond interface language.

Overall, these findings suggest that young people searching in English may encounter a broader and more diverse range of digital mental health resources than users relying solely on native-language search terms. A country-level comparison of English-language versus local-language app search outputs is presented in S2 Table.

### App categories and core characteristics

Based on the information available on the app descriptions provided in the app stores, the 171 included apps were classified into five main categories. A total of 12% (20/171) of apps were classified as content-focused, providing content on mental health with no interactive elements; 50% (85/171) of apps primarily offered self-guided tools like journaling, meditations, and breathing exercises; 16% (28/171) of apps primarily offered an AI-chatbot; 13% (22/171) were gamified apps; and 9% (16/171) of apps provided access to online therapists. An overview of the characteristics of the different app categories is presented in Table 3, and a detailed description of the included apps is provided in S3 Table. It is important to note that across all 171 included apps, only a small subset explicitly named young people as their primary intended audience in the app store description. Apps that explicitly targeted young people included Lyynk (described as designed to help young people better understand themselves, alongside resources for trusted adults), WeCare: Santé mentale (designed for young people aged 18–25 years), and one structured journaling and meditation app specifically designed for young adults and their trusted adults. The vast majority of apps did not specify any youth-focused design intent, which is itself a notable finding given this study’s focus on young people’s digital mental health access. App store age ratings (4+, 12+, 17+/18+) reflect content thresholds applied by platform operators rather than developer-stated target populations, and should not be interpreted as indicators of youth-appropriate design.

**Table 3:** Characteristics of included apps per category.

App characteristics	Structured Journaling and Meditation (n = 85), n (%)	Interactive AI-chatbot (n = 28), n (%)	Access to online therapists (n = 16), n (%)	Content-focused (n = 20), n (%)	Gamified apps (n = 22), n (%)
**Cost**					
Partly Free	71 (84%)	22 (79%)	2 (13%)	17 (85%)	21 (95%)
Fully Free	14 (16%)	6 (21%)	14 (87%)	3 (15%)	1 (5%)
**App store age classification and stated target populations**					
Adults and children	58 (68%)	11 (40%)	6 (37%)	14 (70%)	18 (82%)
Adults and adolescents	23 (27%)	13 (46%)	7 (44%)	5 (25%)	3 (14%)
Adults only	4 (4%)	4 (14%)	3 (19%)	1 (5%)	1 (4%)
**Language availability**					
English only	41 (48%)	18 (64%)	10 (63%)	9 (45%)	9 (41%)
Local language only	3 (4%)	1 (4%)	1 (6%)	2 (10%)	–
English + local language	5 (6%)	–	–	1 (5%)	–
Multilingual (>2 languages)	31 (36%)	6 (21%)	4 (25%)	7 (35%)	12 (54%)

*Note*. Language availability categories were derived from app store metadata. “Local language only” refers to apps available exclusively in one of the languages represented in the included countries (i.e., Croatian, French, Polish, Portuguese, Serbian, or Turkish) without English listed. “English + local language” refers to apps available in English and one local study language only. “Multilingual” refers to apps supporting more than two languages.

#### Content-focused apps.

Content-focused apps were defined and understood as apps that were primarily designed for passive engagement, providing users with access to a range of mental health resources but not incorporating any interactive elements. A total of 12% (20/171) of apps were classified as content-focused. These apps offered four main categories of content: (1) daily affirmations, motivational quotes, and reminders aimed at reframing negative thoughts, enhancing optimism, and supporting self-esteem; (2) educational materials focused on self-confidence, resilience, personal development, and coping strategies; (3) therapy-inspired or evidence-based practices, extending beyond motivational content; and (4) resources linking mental wellbeing to daily routines, including habit tracking and lifestyle choices. The app descriptions stated that most of these apps delivered short-form text (e.g., affirmations, quotes, or summaries) with visual customization, such as backgrounds, themes, and widgets. A subset of apps incorporated audio features (e.g., guided sessions, hypnosis, voice recordings or therapy streams) and a smaller number provided video lessons or tutorials.

Of the content-focused apps, 15% (3/20) were free to download and use, whereas 85% (17/20) were free to download with partial access to features (freemium model). Paid features typically included subscriptions to unlock additional or unlimited content, often described as “premium content.” Other paid options involved customised in-app experiences, such as additional themes, background music, widgets, or personnalised recommendations, as well as ad-free access in apps that otherwise displayed advertisements. In several cases, apps provided a free trial period before requiring a subscription. One app additionally allowed users to make voluntary donations.

Regarding app store age classifications and explicitly stated target populations, 70% (14/20) of the content-focused apps were categorised by the respective App Stores as fit for both adults and children (i.e., assigned an age rating of 4+); with one of these apps recommending parental supervision, and one app explicitly targeted at professionals. Only 5% (1/20) of apps were designated for adults only (assigned an age rating of 18+). Notably, one app marketed as suitable for users aged 13 and older indicated the presence of potentially realistic violence, sexual content or nudity.

Regarding language accessibility, 60% (12/20) of the content-focused apps supported only one language. Of these, 75% (9/12) were available exclusively in English, 17% (2/12) in Portuguese, and 8% (1/12) in Spanish. A further 5% (1/20) of apps offered two languages (English and Portuguese), whereas 35% (7/20) supported multiple languages, ranging from three to thirty-seven languages.

#### Structured journaling and meditation apps.

Structured journaling and meditation apps represented 50% (85/171) of all included apps. These apps provided self-help tools, such as journaling and meditation, without relying heavily on gamification or intensive AI-driven conversational interfaces. These apps facilitated user active engagement through self-help techniques, such as journaling and meditation. While some incorporated AI components for tracking or personalization, their primary purpose was to provide users with interactive tools that support mental health. Among the 85 structured journaling and meditation apps, 16% (14/85) were free to download and use, whereas 84% (71/85) were free to download with partial access to features (freemium model). The latter typically included in-app purchases to remove advertisements or unlock premium content, as well as subscription plans (monthly or annual) or one-time purchases granting lifetime access to app content and resources. Several apps also provided free trials to access premium features, usually for a short period (e.g., one week).

Regarding app store age classifications and explicitly stated target populations, 66% (56/85) of apps were designed for both adults and children (assigned an age rating of 4+). Of these, one app specifically targeted women, one focused on the LGBTQIA community, one was intended exclusively for Catholics, one was designed for young adults and their trusted adults, and one was specifically designed to target young people aged 18–25 years. A further 27% (23/85) of apps were categorised by the App Stores as fit for adults and adolescents (assigned an age rating of 12+); within this group, one app was developed for women only, and another was designed for organizational staff and their family members. The remaining 5% (4/85) of apps were categorised as exclusively for adults (assigned an age rating of 18+), including one accessible only through an employer health plan.

The overall aim of the journaling and meditation apps was to provide users with self-help tools to address a range of mental health–related concerns, such as stress, panic attacks, and mood regulation. Several apps also claimed to address symptoms of depression and anxiety. The most frequently targeted areas were stress, anxiety, sleep problems, and depression. Almost half of these apps provided access to meditation and mindfulness as well as other interactive tools, such as emotion and mood trackers, journaling, and daily check-ins (46%, 39/85). Peer-support community access was available in 1% (1/85) of apps, while another 1% (1/85) claimed to provide scream therapy for stress and anxiety relief. Cognitive Behavioural Therapy (CBT) techniques were incorporated in 4% (3/85) of apps, and 19% (16/85) included sleep-related tools, such as sleep music, sleep stories, sleep trackers, or sleep sounds. Financial and life coaching features were identified in 1% (1/85) of apps, whereas 4% (3/85) included self-discovery tools.

Most journaling and meditation apps were available in a single language (52%, 44/85). Among these, 93% (41/44) were available exclusively in English, 5% (2/44) in French, and 2% (1/44) in Polish. Apps supporting two languages accounted for 12% (10/85), most commonly combining English with French or German. The remaining 36% (31/85) supported more than two languages, ranging from three to thirty-two languages.

#### Interactive AI Chatbot Apps.

Interactive AI chatbot apps were described in their app store listings as employing AI-driven conversational agents to provide mental health support through dialogue-based interactions. According to their descriptions, these apps allow users to interact with virtual agents via conversation, with claimed features including personalised guidance, coping strategies, and emotional support via chat, and in some cases presenting the experience as resembling interactions with a mental health professional. While some interactive AI chatbot apps incorporated additional features, such as mood tracking or journaling, their primary focus was dialogue-based mental health interventions. Among the 28 AI-driven conversational agent apps, 21% (6/28) were entirely free to download and use, whereas 79% (22/28) of apps were free to download with partial access to features (freemium model), typically including in-app purchases, subscription plans, or one-time payments to unlock premium features and additional content.

The main aim of the interactive AI chatbot apps was claiming to offer personalized support, analyse user inputs, detect emotional states or stress levels, and generate contextually appropriate responses. According to app store descriptions, the AI agent was presented as capable of simulating therapeutic conversations, offering coping strategies, providing encouragement, or guiding users through self-help exercises, including those described as based on cognitive-behavioral techniques.

In terms of language availability, 68% (19/28) of apps were available in a single language. Among these, 95% (18/19) were available exclusively in English only and 5% (5/19) in Polish. Apps available in two languages accounted for 11% (3/28), combining English with Ukrainian (33.3%, 1/3), Japanese (33.3% 1/3), or Spanish (33% 1/3). The remaining 21% (6/28) of apps were available in more than two languages, with language availability ranging from four to eighteen languages.

#### Gamified Apps.

Gamified apps include gamification elements to promote positive mental health outcomes. A total of 22 applications integrated gamification strategies, specifically designed to enhance relaxation, reduce stress, and support emotional wellbeing. Of these, 95% (21/22) were free to download with partial access to features (freemium model), typically through in-app purchases, subscription upgrades, or options to remove advertisements, whereas 5% (1/22) of apps were free to download and use. In terms of the target population, 82% (18/22) apps were designed for both adults and children (assigned an age rating of 4+), including those suitable for a broad age range without specific restrictions. Of these, 14% (3/22) targeted adolescents and adults, and 5% (1/22) of apps were directed exclusively toward adult users. (assigned an age rating of 18+).

Regarding core functionality, most apps (82%, 18/22) focused on stress reduction and relaxation through interactive forms of play, such as tactile activities, puzzle-based challenges, or simulation games. Several apps also targeted sleep improvement, mindfulness, and emotional regulation, while a smaller subset claimed to incorporate cognitive-behavioral therapy (CBT) principles or combined mindfulness with breathing exercises. One app delivered daily mental wellness routines and self-care tasks, whereas another employed quiz-based interactions to engage users.

Regarding language availability, 41% (9/22) of gamified apps were offered only in English. A further 5% (1/22) were available in two languages (English and Russian), whereas 54% (12/22) supported multilingual access, ranging from eight to twenty-one languages. The most commonly supported languages included English, French, German, Spanish, Portuguese, Japanese, Korean, and Simplified Chinese.

#### Apps that link to online therapists.

A total of 16 applications function as digital platforms connecting users directly to therapists and facilitating access to professional mental health services. Of these, 87% (14/16) of apps were free to download and use, whereas 13% (2/16) included in-app purchases. Information regarding therapy session costs was provided in 13% (2/16) of apps, and several additionally offered an initial online consultation free of charge.

Regarding app store age classifications and explicitly stated target populations, 44% (7/16) of apps were designed for adults and adolescents, including one specifically aimed at teenagers that required parental consent for registration. A further 37% (6/16) targeted both adults and children (assigned an age rating of 4+), whereas 19% (3/16) were aimed exclusively at adults (assigned an age rating of 18+), with one specifically restricted to employees of partner companies.

Based on app descriptions, 31% (5/16) of apps claimed that therapy was delivered by clinical psychologists, whereas 38% (6/16) stated that services were provided by certified or qualified psychologists. In addition, 38% (6/16) incorporated an initial questionnaire to match users with a suitable therapist, and one of these apps also offered the option to choose between an AI chatbot and a human psychologist.

Most apps linking users to therapists were available in a single language (69%, 11/16), primarily English, with one app available exclusively in Turkish. Apps supporting two languages accounted for 6% (1/16) and offered English and Arabic. The remaining 25% (4/16) supported multiple languages, ranging from three to ten languages.

Some apps were available in languages other than the local study languages, mostly combined with English. For structured journaling and meditation apps, this included English and German (n = 3), English and Russian (n = 1), and English and Ukrainian (n = 1). For interactive AI-chatbot apps, this included English and Ukrainian (n = 1), English and Japanese (n = 1), and English and Spanish (n = 1). One access-to-online-therapists app was available in English and Arabic only. One content-focused app was available exclusively in Spanish. One gamified app was available in English and Russian only.

## Discussion

### Principal findings

Mental-health-related smartphone applications have become increasingly prevalent, and prior research has evaluated such apps in various countries, including China [27,28], India [29], and France [22]. Previous reviews have also examined the mental-health app landscape across multiple countries within a single analysis [23]. However, to our knowledge, this is the first review to synthesize the full range of available and widely used mental health-related applications that young people might frequently encounter across seven European countries: Croatia, France, Ireland, Poland, Portugal, Serbia, and Türkiye. Accordingly, this review aimed to identify cross-country differences and analyze the content of these applications using available app store data, with a particular focus on store descriptions.

The findings suggest that language plays a critical role in shaping the visibility and presentation of mental health-related applications across countries and app stores. Importantly, the language used to conduct searches directly influenced which apps were returned, with English-language searches consistently yielding a broader range of popular commercialised applications, such as *Headspace*, *BetterMe*, and *Daylio*, than local language searches. This pattern underscores the global dominance of English-language apps and suggests limited localisation or cultural adaptation. Although application popularity is often inferred from user ratings, these ratings are inherently biased, reflecting the experiences of particular user groups rather than the clinical effectiveness of the applications [30]. Substantial empirical evidence supports the efficacy of Headspace in promoting psychological wellbeing [23,30–33], which may partly account for its strong market presence and visibility. However, given that the studies cited were published after the launch of Headspace (2022–2024 vs. 2012, respectively), it could be argued that its early market dominance may have, in fact, simulated subsequent empirical research on its effectiveness. In contrast, the majority of other top-ranked applications lack empirical validation, including evidence from randomized controlled trials, which raises concerns about the potential risks associated with unregulated or non-evidence-based mental health-related apps, particularly for young people seeking help. This issue has been widely discussed in the literature [4,5,34].

In contrast, searches conducted in the native language often returned fewer results or more localized results, some of which were irrelevant to the study’s objectives. For example, Turkish search terms, such as *“psikolojik iyi oluş”* (psychological wellbeing), produced fewer results overall than equivalent English terms across both app stores. This might suggest that clinical terminology is less commonly used in local app descriptions, which limits the visibility of such apps in native-language searches. Moreover, cross-country differences were observed in app store data, particularly in ratings and download counts, highlighting inconsistencies in how mental health apps are presented in the same app stores depending on the geolocation of the search. This further accentuates differences across linguistic and regional contexts, as shown in the results section. Collectively, these findings suggest that young people searching in their native languages may encounter a narrower and more commercialized selection of apps, compared to the broader and more clinically diverse range available through English-language searches, potentially representing an accessibility barrier for non-English-speaking youth. This is especially important since expressing mental health concerns is easier in one’s native language than a foreign language [35].

During the app selection process, the primary reason for exclusion was that most applications had not been updated within the six months before data collection. This may indicate that apps remaining outdated for extended periods tend to be of lower quality, particularly in terms of privacy protections, feature relevance, and evidence base [36]. Additionally, low user retention reduces developers’ incentives to maintain or update their applications. Mental health applications are characterized by particularly low engagement rates; for example, a study of 93 mental health apps reported a median 15-day retention rate of 3.9% and a 30-day retention rate of 3.3%, suggesting that most users discontinue use within the first two weeks [37]. As user activity declines, developers may see little reason to continue releasing regular updates. Consequently, many apps become effectively “finished” from the publisher’s perspective, remaining available without active development. This reflects a broader “build fast, move on” model within the digital marketplace, in which developers prioritise rapid release and short-term user growth over sustained maintenance or evidence-based refinement [38].

Our review also found that meditation and journaling applications constitute the majority of currently available mental health-related apps in the app stores. Searches for mindfulness-based applications have increased significantly in recent years, reflecting their dominance in the digital mental health marketplace [3,38]. Moreover, mindfulness applications have demonstrated effectiveness in reducing symptoms of anxiety and depression and enhancing psychological wellbeing [39], which may help explain their widespread popularity and visibility. These findings suggest that the visibility of mental health apps is associated not only with linguistic accessibility but also with applications’ content focus, with mindfulness and journaling apps more likely to surface in app store searches. However, most of these applications are oriented toward general mental wellbeing rather than evidence-based therapeutic care [40], which may limit their suitability for young people seeking more clinically guided support.

A more detailed analysis revealed that the majority of the mental health-related apps were not entirely free to use. Most offered a freemium model, offering a basic version at no cost alongside premium features through subscription or one-time payment. This model allows young people to explore a wide range of mental health tools without financial barriers, use these apps as an adjunct to ongoing therapy, or as an alternative for those who prefer self-management of symptoms [40]. Notably, although most apps connecting users to online therapists were technically free to download, this did not necessarily imply free access to therapy itself. In many cases, app descriptions indicated that consultations, subscriptions, employer-based healthcare access, or session fees were required to access professional services. Therefore, “free download” classifications should not be interpreted as equivalent to free mental health care access.

However, freemium models also restrict access to key features or evidence-based modules, potentially pressuring vulnerable youth to download unhelpful or costly apps and undermining both effectiveness and user trust [41]. These accessibility dynamics may further explain why meditation and journaling applications dominate the marketplace, as their free content enables engagement with mental health support even when premium features remain inaccessible.

Taken together, the patterns identified in this review reflect not just individual app characteristics but systemic features of the digital mental health ecosystem that have direct implications for governance, equity, and implementation. The concentration of visible apps in English, combined with limited localisation, points to a structural gap in how digital health tools are developed and regulated: market incentives favour scale and English-speaking audiences over cultural or linguistic appropriateness. For instance, Muñoz, Camacho, and Torous [42] found that only 14.5% of mental health apps on the commercial marketplace were operable in Spanish, despite Spanish being the fourth most spoken language globally, a disparity that can reasonably be extrapolated with far fewer speakers. Additionally, Whitehead et al. [43] highlighted language and culture as potential barriers to the accessibility of digital health interventions. From a governance perspective, this underscores the absence of coordinated regulatory frameworks that could ensure equitable app visibility and quality across national app stores.

Current app store curation relies largely on commercial marketplace dynamics rather than clinical or public health criteria; app visibility is driven by popularity metrics such as downloads and ratings, which have been shown not to correlate with evidence-based quality or privacy standards [44], meaning that the apps most visible to young people in any given country are shaped more by commercial success than by evidence of effectiveness or cultural fit. Furthermore, the prevalence of freemium cost models introduces tiered access structure within digital mental health ecosystems, where key features are often restricted behind paywalls, raising concerns about equitable access to effective support [3]. This has implications for equity, particularly for young people who may rely on free resources. In the absence of standardized regulatory frameworks or certification systems across countries, users and healthcare systems are left to navigate a highly heterogeneous and largely unregulated marketplace. These findings underscore the need for coordinated policy efforts at national and international levels to ensure that digital mental health interventions are accessible, evidence-based, and culturally appropriate, particularly for youth populations.

### Limitations and directions for future research

While the current study reveals important cross-country similarities and discrepancies in the availability of mental health smartphone apps, the findings should be interpreted in light of its limitations. First, relevant apps may have been missed in the identification process. App identification relied on the algorithms of the Apple App Store and Google Play Store, which vary according to geolocation and language, potentially affecting app capture and ranking. The search strategy captured only the first 10 results per keyword in seven European countries, using English and local languages. Although this approach reflects real-world behaviour, it may not fully represent the apps most commonly used by young people. Moreover, some app store entries lacked key information, such as downloads or supported languages, and inconsistencies in app naming may have led to relevant apps being missed.

The analysis was based on publicly available descriptions rather than direct testing of the apps. As such, app functionality, gamified features, AI integration, or therapist credentials could not be verified. Additionally, while this study examined which apps were accessible and visible to young people within commercial app stores, it did not include a qualitative assessment of app interface design, developmental appropriateness, readability, visual presentation, or intervention suitability for youth populations. As such, the findings should not be interpreted as evidence that apps categorised as “4+” or “12+” were specifically designed for or appropriate for children or adolescents. Future research should incorporate direct app testing and qualitative evaluation methods to assess the extent to which digital mental health applications are genuinely youth-centred in their design, content, and therapeutic approach.

Likewise, apps were categorised by thematic group and described by cost, target population, and language availability; however, no formal quality assessment [e.g., the Mobile App Rating Scale, [45]] was conducted. To gain a better understanding of which mental health applications young people use, how they discover them, and which features they perceive as most beneficial or insufficient, future research should actively engage young people from diverse cultural and national contexts.

Relatedly, while the current study compares app features, it does not examine user responses, such as number of downloads or user ratings. Although user ratings and download indicators were extracted during data collection, they were not systematically analysed as comparative variables in this study. User ratings reflect subjective experiences of self-selected users and may vary across country-specific app store listings, limiting their reliability for cross-country comparison. Furthermore, download data are not consistently reported across platforms: the Apple App Store does not provide download counts, while the Google Play Store reports downloads in broad ranges rather than exact figures. Download data also does not reflect actual usage of the apps. As such, these metrics were not suitable for robust comparative analysis. Future research could build on this work by incorporating more precise and standardised measures of user engagement and adoption to better understand the real-world reach and impact of digital mental health applications.

A further limitation concerns the geographic scope of the present analysis. The seven countries were selected through purposive sampling to capture heterogeneity across European geographic regions, political, and health system contexts, and linguistic environments, rather than as a representative sample of all European contexts. As such, the findings should be interpreted as illustrative of cross-country patterns in digital mental health app visibility rather than generalised to Europe as a whole. Furthermore, the sample does not include non-European English-language markets such as the United States, which represents one of the largest and most commercially dominant markets for digital mental health applications [19]. Extending this comparative framework to North American or other non-European app store contexts is an important direction for future research. Additionally, the present analysis relied exclusively on app store descriptions and metadata, which do not incorporate population-level variables such as economic status or age structure. The countries included in this study span settings with varying economic and demographic profiles. A more systematic integration of socioeconomic and demographic data would substantially strengthen the interpretive power of cross-country comparisons and is an important direction for future research. App developer country of origin was not systematically extracted because this information was inconsistently available across app store platforms and developer profiles. Consequently, the present study was unable to assess whether apps were developed locally or internationally, or the extent to which app origin may have influenced linguistic adaptation, cultural relevance, or localisation practices. Given the strong dominance of English-language commercial applications observed across countries in the present review, future research should examine developer origin and regional development patterns in greater detail to better understand how digital mental health resources are distributed, localised, and adapted across different cultural and linguistic contexts.

Finally, the expanding integration of AI within mental health applications warrants critical examination to distinguish genuine technological innovation from marketing rhetoric and to evaluate how AI influences user trust, app selection, and engagement. Attention should also be directed toward app developers and their professional credentials, as these factors may affect perceptions of reliability, content quality, and user confidence. Collectively, these considerations underscore the potential value of establishing a globally recognized certification framework for digital mental health applications, promoting safety, evidence-based practice, and ethical design.

## Conclusion

To our knowledge, this study provides the first cross-country and cross-language mapping of mental health-related applications accessible to young people across seven European countries. Our systematic search of the Apple App Store and Google Play Store revealed that the majority of available mental health apps are commercially driven, English-language freemium models, primarily focused on meditation and journaling. Local-language or culturally adapted alternatives were scarce, highlighting persistent inequities in accessibility for non-English speaking youth. To promote equitable access, future prevention and intervention initiatives should prioritise the development and evaluation of culturally relevant, evidence-based, and linguistically inclusive digital mental health interventions.

## Supporting information

S1 Checklist PRISMA 2020 checklist. Adapted from the Preferred Reporting Items for Systematic reviews and Meta-Analyses (PRISMA) 2020 Statement, licensed under the Creative Commons Attribution 4.0 International (CC BY 4.0) License.
(DOCX)

S1 Table Output of the initial app store searches per country for each of the four keywords (“mental health,” “psychological wellbeing,” “self-help,” and “stress”), presented separately for English-language and local-language searches across all seven countries (Croatia, France, Ireland, Poland, Portugal, Serbia, and Türkiye) and both app stores (Apple App Store and Google Play Store). (DOCX)

S2 Table Comparative overview of app store search results across the seven included countries (Croatia, France, Ireland, Poland, Portugal, Serbia, and Türkiye). For each country, the following information is provided: English-language search patterns, local-language search patterns, apps explicitly targeting young people identified in searches, and the dominant app types identified.
(DOCX)

S3 Table Detailed description of all 171 included apps, organised by app category (Structured Journaling & Meditation Apps, Interactive AI-chatbot Apps, Apps Providing Access to Online Therapists, Content-Focused Apps, and Gamified Apps). For each app, the following information is provided: app name, overview of aims, key features, language availability, target population (based on app store age rating), and cost model.
(DOCX)

## References

[1] World Health Organization. World mental health report: transforming mental health for all. Geneva: World Health Organization; 2022. Available from: https://www.who.int/publications/i/item/9789240049338

[2] Liverpool S, Mc Donagh C, Feather J, Uzondu C, Howarth M, Bannerman F, et al. Updates on digital mental health interventions for children and young people: systematic overview of reviews. Eur Child Adolesc Psychiatry. 2025;34(10):2961–74. doi: 10.1007/s00787-025-02722-9 40278894 PMC12592321

[3] Torous J, Linardon J, Goldberg SB, Sun S, Bell I, Nicholas J, et al. The evolving field of digital mental health: current evidence and implementation issues for smartphone apps, generative artificial intelligence, and virtual reality. World Psychiatry. 2025;24(2):156–74. doi: 10.1002/wps.21299 40371757 PMC12079407

[4] Koh J, Tng GYQ, Hartanto A. Potential and pitfalls of mobile mental health apps in traditional treatment: an umbrella review. J Pers Med. 2022;12(9):1376. doi: 10.3390/jpm12091376 36143161 PMC9505389

[5] Lau N, O’Daffer A, Colt S, Yi-Frazier JP, Palermo TM, McCauley E, et al. Android and iPhone mobile apps for psychosocial wellness and stress management: systematic search in app stores and literature review. JMIR Mhealth Uhealth. 2020;8(5):e17798. doi: 10.2196/17798 32357125 PMC7275252

[6] Camacho E, Cohen A, Torous J. Assessment of mental health services available through smartphone apps. JAMA Netw Open. 2022;5(12):e2248784. doi: 10.1001/jamanetworkopen.2022.48784 36576737 PMC9857226

[7] Bell IH, Thompson A, Valentine L, Adams S, Alvarez-Jimenez M, Nicholas J. Ownership, use of, and interest in digital mental health technologies among clinicians and young people across a spectrum of clinical care needs: cross-sectional survey. JMIR Ment Health. 2022;9(5):e30716. doi: 10.2196/30716 35544295 PMC9133993

[8] Pretorius C, Coyle D. Young people’s use of digital tools to support their mental health during covid-19 restrictions. Front Digit Health. 2021;3:763876. doi: 10.3389/fdgth.2021.763876 34927133 PMC8671300

[9] Rickwood D, Deane FP, Wilson CJ, Ciarrochi J. Young people’s help-seeking for mental health problems. Aust E-J Adv Ment Health. 2005;4(3):218–51. doi: 10.5172/jamh.4.3.218

[10] Gulliver A, Griffiths KM, Christensen H. Perceived barriers and facilitators to mental health help-seeking in young people: a systematic review. BMC Psychiatry. 2010;10:113. doi: 10.1186/1471-244X-10-113 21192795 PMC3022639

[11] Pretorius C, Chambers D, Coyle D. Young people’s online help-seeking and mental health difficulties: systematic narrative review. J Med Internet Res. 2019;21(11):e13873. doi: 10.2196/13873 31742562 PMC6891826

[12] Balaskas A, Schueller SM, Cox AL, Doherty G. The functionality of mobile apps for anxiety: systematic search and analysis of engagement and tailoring features. JMIR Mhealth Uhealth. 2021;9(10):e26712. doi: 10.2196/26712 34612833 PMC8529472

[13] Alqahtani F, Orji R. Insights from user reviews to improve mental health apps. Health Informatics J. 2020;26(3):2042–66. doi: 10.1177/1460458219896492 31920160

[14] Thach KS. User’s perception on mental health applications: a qualitative analysis of user reviews. In: 2018 5th NAFOSTED Conference on Information and Computer Science (NICS) [Internet]. 2018. pp. 47–52. [cited 2026 Mar 31]. Available from: https://ieeexplore.ieee.org/document/8606901

[15] Huguet A, Rao S, McGrath PJ, Wozney L, Wheaton M, Conrod J, et al. A systematic review of cognitive behavioral therapy and behavioral activation apps for depression. PLoS One. 2016;11(5):e0154248. doi: 10.1371/journal.pone.0154248 27135410 PMC4852920

[16] Ben-Zeev D, Kaiser SM, Brenner CJ, Begale M, Duffecy J, Mohr DC. Development and usability testing of FOCUS: a smartphone system for self-management of schizophrenia. Psychiatr Rehabil J. 2013;36(4):289–96. doi: 10.1037/prj0000019 24015913 PMC4357360

[17] Newton A, Bagnell A, Rosychuk R, Duguay J, Wozney L, Huguet A, et al. A Mobile Phone-Based App for Use During Cognitive Behavioral Therapy for Adolescents With Anxiety (MindClimb): User-Centered Design and Usability Study. JMIR Mhealth Uhealth. 2020;8(12):e18439. doi: 10.2196/18439 33289671 PMC7755529

[18] Hopkin G, Coole H, Edelmann F, Ayiku L, Branson R, Campbell P, et al. Toward a new conceptual framework for digital mental health technologies: scoping review. JMIR Ment Health. 2025;12:e63484. doi: 10.2196/63484 39969824 PMC11864090

[19] Robinson A, Flom M, Forman-Hoffman VL, Histon T, Levy M, Darcy A, et al. Equity in digital mental health interventions in the United States: where to next? J Med Internet Res. 2024 Sep 24;26:e59939. doi: 10.2196/59939 39316436 PMC11462105

[20] Fürtjes S, Gebel E, Kische H, Beesdo-Baum K. Characteristic of mental health app usage: a cross-sectional survey in the general population. BMC Public Health. 2024;24(1):3133. doi: 10.1186/s12889-024-20500-1 39533246 PMC11555976

[21] Báez Gutiérrez N, Rodríguez Ramallo H, Fernández González M, Abdel-Kader Martín L. Smartphone apps for patients with hematologic malignancies: systematic review and evaluation of content. JMIR Mhealth Uhealth. 2022;10(9):e35851. doi: 10.2196/35851 36125860 PMC9533204

[22] Carrouel F, du Sartz de Vigneulles B, Bourgeois D, Kabuth B, Baltenneck N, Nusbaum F, et al. Mental health mobile apps in the French App store: assessment study of functionality and quality. JMIR Mhealth Uhealth. 2022;10(10):e41282. doi: 10.2196/41282 36223178 PMC9607929

[23] Eis S, Solà-Morales O, Duarte-Díaz A, Vidal-Alaball J, Perestelo-Pérez L, Robles N, et al. Mobile applications in mood disorders and mental health: systematic search in Apple app store and google play store and review of the literature. Int J Environ Res Public Health. 2022;19(4):2186. doi: 10.3390/ijerph19042186 35206373 PMC8871536

[24] Larsen ME, Nicholas J, Christensen H. Quantifying app store dynamics: longitudinal tracking of mental health apps. JMIR MHealth UHealth. 2016;4(3):e96. doi: 10.2196/mhealth.6020 27507641 PMC4995352

[25] Donker T, Petrie K, Proudfoot J, Clarke J, Birch M-R, Christensen H. Smartphones for smarter delivery of mental health programs: a systematic review. J Med Internet Res. 2013;15(11):e247. doi: 10.2196/jmir.2791 24240579 PMC3841358

[26] McGorry PD, Mei C, Dalal N, Alvarez-Jimenez M, Blakemore S-J, Browne V, et al. The Lancet Psychiatry Commission on youth mental health. Lancet Psychiatry. 2024;11(9):731–74. doi: 10.1016/S2215-0366(24)00163-9 39147461

[27] Yin H, Wardenaar KJ, Wang Y, Wang N, Chen W, Zhang Y, et al. Mobile mental health apps in china: systematic app store search. J Med Internet Res. 2020;22(7):e14915. doi: 10.2196/14915 32716301 PMC7418006

[28] Wu X, Xu L, Li P, Tang T, Huang C. Multipurpose mobile apps for mental health in Chinese App stores: content analysis and quality evaluation. JMIR MHealth UHealth. 2022 Jan 4;10(1):e34054. doi: 10.2196/34054 34982717 PMC8767465

[29] Mehrotra S, Tripathi R, Sengupta P, Karishiddimath A, Francis A, Sharma P, et al. Evaluating characteristics and quality of mental health apps available in app stores for indian users: systematic app search and review. JMIR Mhealth Uhealth. 2025;13:e79238. doi: 10.2196/79238 41004798 PMC12514412

[30] Neary M, Schueller SM. State of the field of mental health apps. Cogn Behav Pract. 2018;25(4):531–7. doi: 10.1016/j.cbpra.2018.01.002 33100810 PMC7584258

[31] O’Daffer A, Colt SF, Wasil AR, Lau N. Efficacy and conflicts of interest in randomized controlled trials evaluating headspace and calm apps: systematic review. JMIR Ment Health. 2022;9(9):e40924. doi: 10.2196/40924 36125880 PMC9533203

[32] Callahan C, Kimber J, Hu E, Tanner L, Kunkle S. The real-world impact of app-based mindfulness on headspace members with moderate and severe perceived stress: observational study. JMIR Mhealth Uhealth. 2024;12:e52968. doi: 10.2196/52968 38488513 PMC10986332

[33] Pierce ME, Mirabito G, Verhaeghen P. Mind the app: more time spent on headspace leads to beneficial day-to-day changes in mindfulness, depression, anxiety and stress in college students. Cogent Ment Health. 2024;3(1):2400878. doi: 10.1080/28324765.2024.2400878 41262665 PMC12442982

[34] Coulon SM, Monroe CM, West DS. A systematic, multi-domain review of mobile smartphone apps for evidence-based stress management. Am J Prev Med. 2016;51(1):95–105. doi: 10.1016/j.amepre.2016.01.026 26993534

[35] Ivaz L, Costa A, Duñabeitia JA. The emotional impact of being myself: Emotions and foreign-language processing. J Exp Psychol Learn Mem Cogn. 2016;42(3):489–96. doi: 10.1037/xlm0000179 26348199

[36] Stoeckl SE, Torres-Hernandez E, Camacho E, Torous J. Assessing the Dynamics of the Mental Health Apple and Android App Marketplaces. J Technol Behav Sci. 2023;1–8. doi: 10.1007/s41347-023-00300-x 36712910 PMC9873536

[37] Baumel A, Muench F, Edan S, Kane JM. Objective user engagement with mental health apps: systematic search and panel-based usage analysis. J Med Internet Res. 2019;21(9):e14567. doi: 10.2196/14567 31573916 PMC6785720

[38] Mani M, Kavanagh DJ, Hides L, Stoyanov SR. Review and evaluation of mindfulness-based iPhone Apps. JMIR Mhealth Uhealth. 2015;3(3):e82. doi: 10.2196/mhealth.4328 26290327 PMC4705029

[39] Gál É, Ștefan S, Cristea IA. The efficacy of mindfulness meditation apps in enhancing users’ well-being and mental health related outcomes: a meta-analysis of randomized controlled trials. J Affect Disord. 2021;279:131–42. doi: 10.1016/j.jad.2020.09.134 33049431

[40] Wasil AR, Palermo E, Lorenzo Luaces L, DeRubeis RJ. Is there an app for that? A review of popular apps for depression, anxiety, and well being. Cogn Behav Pract. 2022;29(3):246–58. doi: 10.1016/j.cbpra.2021.10.001

[41] Eagle T, Mehrotra A, Sharma A, Zuniga A, Whittaker S. “Money Doesn’t Buy You Happiness”: negative consequences of using the freemium model for mental health apps. Proc ACM Hum‑Comput Interact. 2022;6(CSCW2). doi: 10.1145/3555155

[42] Muñoz AO, Camacho E, Torous J. Marketplace and literature review of spanish language mental health apps. Front Digit Health. 2021;3:615366. doi: 10.3389/fdgth.2021.615366 34713093 PMC8521936

[43] Whitehead L, Talevski J, Fatehi F, Beauchamp A. Barriers to and facilitators of digital health among culturally and linguistically diverse populations: qualitative systematic review. J Med Internet Res. 2023;25:e42719. doi: 10.2196/42719 36853742 PMC10015358

[44] Sezgin E. Can we use commercial mobile apps instead of research mobile apps in healthcare research? Front Public Health. 2021;9:685439. doi: 10.3389/fpubh.2021.685439 34368058 PMC8342752

[45] Stoyanov SR, Hides L, Kavanagh DJ, Zelenko O, Tjondronegoro D, Mani M. Mobile app rating scale: a new tool for assessing the quality of health mobile apps. JMIR Mhealth Uhealth. 2015;3(1):e27. doi: 10.2196/mhealth.3422 25760773 PMC4376132

