# Supplementary material for: Digital mental health apps for young people in Europe: A systematic cross-country analysis
Source: PLOS Digit Health. 2026 Aug 27;5(8):e0001622. doi: 10.1371/journal.pdig.0001622 (PMC13521388; doi:10.1371/journal.pdig.0001622)
Supplement: Supplementary file 1 [file pdig.0001622.s006.pdf]

Response to reviewers

We thank the editor and reviewers sincerely for their positive response to our manuscript and their recommendation about how the manuscript could be further improved. We have carefully addressed all comments and provided point-by-point responses below, indicating the corresponding line and page numbers where changes have been made in the revised manuscript. Additionally, we have summarized these changes in our response.

Reviewer #1:

1. **A key limitation is that while the authors compare app features, they do not examine user responses, such as number of downloads or user ratings, which could provide additional insight into app adoption and impact.**

   Response. We thank the reviewer for this important observation. We wish to clarify that user ratings and download indicators were collected. However, we did not use them as comparative metrics across countries or app categories, because they lack the precision and consistency required for a meaningful statistical analysis. User ratings reflect the experiences of self-selected user groups and vary substantially across country-specific app store listings, a variation that is shaped by differences in local user bases, cultural norms of rating behaviour, and platform dynamics. Moreover, the Apple App Store does not provide download counts, while the Google Play Store provides download ranges rather than exact figures.

   In response to this comment, we have integrated this limitation into the second limitation paragraph, recognising it as a direction for future research. See Page 24, Lines 3-12:
   "Relatedly, while the current study compares app features, it does not examine user responses, such as number of downloads or user ratings. Although user ratings and download indicators were extracted during data collection, we were not able to systematically analyse them as comparative variables in this study. User ratings reflect subjective experiences of self-selected users and may vary across country-specific app store listings, limiting their reliability for cross-country comparison. Furthermore, download data are not consistently reported across platforms: the Apple App Store does not provide download counts, while the Google Play Store reports downloads in broad ranges rather than exact figures. As such, these metrics were not suitable for robust comparative analysis. Future research could build on this work by incorporating more

precise and standardised measures of user engagement and adoption to better understand the real-world reach and impact of digital mental health applications."

2. **The authors may also consider exploring differences by country and, if possible, including the United States or North America, where English is also widely used. In addition, factors such as economic status and population age structure may be relevant and worth considering.**

   Response. We thank the reviewer for this suggestion. We agree that comparisons with non-European English-language markets (such as the US) would be particularly informative. However, expanding the study to include these additional regions is beyond the scope of the present analysis. We have added this recommendation explicitly to the Limitations and Directions for Future Research section.

   Regarding economic status and population age structure, we note that the current analysis was based solely on app store descriptions and metadata, which do not include population-level variables. We acknowledge this as a limitation of the app store-derived data approach and have noted in the revised manuscript that the country selection spans settings with varying economic contexts, while recognising that a more systematic integration of socioeconomic and demographic factors would strengthen future cross-country comparisons. See Page 24, Lines 13-27:

   "A further limitation concerns the geographic scope of the present analysis. The seven countries were selected through purposive sampling to capture heterogeneity across European geographic regions, political, and health system contexts, and linguistic environments, rather than as a representative sample of all European contexts. As such, the findings should be interpreted as illustrative of cross-country patterns in digital mental health app visibility rather than generalised to Europe as a whole. Furthermore, the sample does not include non-European English-language markets such as the United States, which represents one of the largest and most commercially dominant markets for digital mental health applications [19]. Extending this comparative framework to North American or other non-European app store contexts is an important direction for future research. Additionally, the present analysis relied exclusively on app store descriptions and metadata, which do not incorporate population-level variables such as economic status or age structure. The countries included in this study span settings with varying economic and demographic profiles. A more systematic integration of socioeconomic and

demographic data would substantially strengthen the interpretive power of cross-country comparisons and is an important direction for future research..”

<u>Reviewer #2:</u> This manuscript presents a systematic, cross-country descriptive analysis of mental health–related smartphone applications identified through app store searches conducted across seven European countries and languages. The study addresses an important and timely question concerning linguistic accessibility, commercialization, and the visibility of digital mental health resources encountered by young people in real-world app marketplaces. The methods are clearly reported and appropriate for the descriptive objectives of the study. However, several interpretive statements extend beyond what can be supported by app store–derived data alone, and the manuscript would benefit from clearer justification of scope, improved consistency in reporting results, and stronger articulation of how the findings inform digital health systems, governance, and equity. I therefore recommend major revision.

1. **Justification of Country Selection and Scope. Section: Methods. The title frames the analysis as representing “Europe,” yet only seven countries are included. While this selection may be pragmatic or data-driven, the rationale for how these countries collectively represent Europe is not clearly articulated. Recommendation: Please explicitly justify the selection of the seven countries (e.g., geographic spread, linguistic diversity, population size, or app store relevance) and clarify whether findings should be interpreted as illustrative rather than representative of Europe as a whole. This clarification is important to avoid overgeneralization.**

   Response. We thank the reviewer for raising this important point. We have revised the country selection rationale in the Methods section to frame the seven counties (Croatia, France, Ireland, Poland, Portugal, Serbia, and Türkiye) as a purposive sample selected to capture heterogeneity, rather than as a researcher-convenience sample.

   Specifically, the revised text emphasises that the countries were chosen to reflect diversity in geographic regions (Western, Southern, and Central/Eastern Europe), political and health system contexts, and linguistic and cultural environments. We also note that the research team prioritised countries where members had sufficient linguistic and contextual expertise to systematically search, screen, and interpret app content in the local language(s). We have explicitly clarified that the seven countries constitute an illustrative rather than representative sample of European contexts.See Page 5, Lines 9-14:
   “This study focuses on seven European countries (Croatia, France, Ireland, Poland,

Portugal, Serbia, and Türkiye) selected through purposive sampling to capture heterogeneity rather than to provide exhaustive coverage of all European countries. The countries were chosen to reflect diversity in geographic regions (Western, Southern, and Central/Eastern Europe), political and health system contexts, and linguistic and cultural environments. We also prioritised countries where the research team had sufficient linguistic and contextual expertise to systematically search, screen, and interpret app content in the local language(s). Consequently, the seven countries should be understood as an illustrative sample of European contexts, rather than a representative selection of all European countries."

2. **Alignment Between Data Source and Interpretation. Sections: Results / Discussion. Several statements imply provision, verification, or effectiveness of therapeutic content (e.g., CBT-based interventions, AI-driven personalised support, professional involvement), despite the analysis relying solely on app store descriptions. Examples of wording patterns that require caution include: • Claims that apps provide CBT or therapy, rather than claiming to do so. • Statements that AI chatbots deliver personalised mental health support, rather than being described as AI-based. • References to clinical or professional involvement without clarification that this is based on developer claims. • Language implying user benefit or quality (e.g., apps support mental health needs). Recommendation: Please review the Results and Discussion to ensure consistent, cautious phrasing throughout, clearly indicating that therapeutic approaches, AI functionality, professional input, and intended benefits are marketed or claimed features, not independently verified functionality. This alignment is essential to ensure conclusions remain fully supported by the data source.**

   Response. We especially thank the reviewer for this very important comment. The manuscript has been carefully reviewed to ensure that all descriptions of therapeutic approaches, AI functionality, professional involvement, and intended benefits are clearly framed as claims made in app store descriptions rather than independently verified features. Specifically: (1) references to apps providing CBT have been reworded to "claimed to incorporate CBT-informed principles"; (2) descriptions of AI chatbot functionality now explicitly state these are developer-reported features based on app store descriptions, not independently verified; (3) claims regarding clinical psychologist or qualified therapist involvement are now described as "developer-stated claims based solely on app store descriptions"; and (4) broader language implying user benefit has been

qualified throughout with phrases such as "according to app store descriptions" and "claimed features." We believe these revisions bring the manuscript into full alignment with the descriptive scope of the data.

3. **Framing Within Digital Health Systems. Section: Discussion. While the Discussion accurately summarizes the findings, it remains largely descriptive and focused on individual app characteristics. For a PLOS Digital Health readership, clearer system-level interpretation would strengthen the manuscript. Recommendation: Please strengthen the Discussion to clarify how observed patterns—such as English-language dominance, freemium cost models, and app store visibility—inform understanding of digital health governance, equity, regulation, or implementation for youth mental health services, while remaining within the descriptive scope of the data.**

   Response. We thank the reviewer for this helpful suggestion. We agree that the Discussion benefited from stronger system-level framing. We have added a paragraph to the discussion that interprets the observed patterns (English-language dominance, freemium models, and app store visibility) in terms of their implications for digital health governance, equity, regulation, and implementation for youth mental health services. Specifically, we now discuss how commercial app store algorithms, rather than clinical or public health criteria, currently shape which apps are most visible to young people, and highlight the need for policy-level responses to support culturally and linguistically inclusive digital mental health ecosystems. This addition remains grounded in the descriptive scope of our data while offering the interpretive layer appropriate for a PLOS Digital Health readership. See Page 22, Line 9-25; Page 23, Lines 1-3:

   "Taken together, the patterns identified in this review reflect not just individual app characteristics but systemic features of the digital mental health ecosystem that have direct implications for governance, equity, and implementation. The concentration of visible apps in English, combined with limited localisation, points to a structural gap in how digital health tools are developed and regulated: market incentives favour scale and English-speaking audiences over cultural or linguistic appropriateness. For instance, Muños, Camacho, and Torous [42] found that only 14.5% of mental health apps on the commercial marketplace were operable in Spanish, despite Spanish being the fourth most spoken language globally, a disparity likely to be even greater in languages with smaller speaker populations. Additionally, Whitehead et al. [43] highlighted language and culture

as potential barriers to the accessibility of digital health interventions. From a governance perspective, this underscores the absence of coordinated regulatory frameworks that could ensure equitable app visibility and quality across national app stores.

Current app store curation relies largely on commercial marketplace dynamics rather than clinical or public health criteria; app visibility is driven by popularity metrics such as downloads and ratings, which have been shown not to correlate with evidence-based quality or privacy standards [44], meaning that the apps most visible to young people in any given country are shaped more by commercial success than by evidence of effectiveness or cultural fit. Furthermore, the prevalence of freemium cost models introduces tiered access structure within digital mental health ecosystems, where key features are often restricted behind paywalls, raising concerns about equitable access to effective support [3]. This has implications for equity, particularly for young people who may rely on free resources. In the absence of standardized regulatory frameworks or certification systems across countries, users and healthcare systems are left to navigate a highly heterogeneous and largely unregulated marketplace. These findings underscore the need for coordinated policy efforts at national and international levels to ensure that digital mental health interventions are accessible, evidence-based, and culturally appropriate, particularly for youth populations."

4. **Definition and Use of the Term "Young People". Sections: Methods / Results. The manuscript frequently refers to apps targeting "young people," although many identified apps appear to be rated for broad age groups rather than explicitly designed for adolescents. In particular, the phrase "young adults and their trusted adults" requires clarification to confirm whether this wording is accurate and how it aligns with the study's conceptualization of "young people." Recommendation: Please clarify how "young people" is operationalized in this study and reflect on how app store age ratings and target descriptions affect interpretation, including implications for youth appropriateness and safeguarding.**

   Response. We thank the reviewer for highlighting this important conceptual issue. We have added clarification to the Methods section specifying that "young people" in this study is defined as individuals aged 11-25 years, a range that captures the key developmental transitions from childhood to adolescence and from adolescence to early adulthood, during which the majority of mental health difficulties emerge (McGorry et al., 2024). Therefore, this age range reflects the definition adopted by the YouthDMH COST Action and is grounded in a broader evidence base. We acknowledge that app store

age ratings (4+, 12+, 18+) reflect platform content guidelines rather than intended target populations, and that these do not map straightforwardly onto our conceptualisation of young people. Where app store descriptions referred explicitly to a target population (e.g., "young adults and their trusted adults," or "specifically designed for young people aged 18–25"), we recorded this language verbatim from the store listing, as these descriptions represent the developers' stated intent rather than our own categorisation. See Page 6, Lines 9-15:

"In this study, "young people" was defined as individuals aged 11-25. This age range captures the key developmental transitions from childhood to adolescence and from adolescence to early adulthood, during which the majority of mental health difficulties emerge: 75% of mental health conditions have their onsent before the age of 25 (McGorry et al., 2024). It is important to note that app store classifications do not typically align with this definition. Instead, apps are categorised using broad age ratings (e.g., 4+, 12+, 18+), which do not necessarily reflect developmental appropriateness or suitability for youth mental health support. Therefore, age-related classifications reported in this study reflect app store metadata rather than verified target populations."

5. **Abbreviations. Please ensure that all abbreviations are introduced at first use. For example, the abbreviation "VOS" appears without prior definition.**

   Response. We thank the reviewer for noting this. All abbreviations have now been checked and defined at their first occurrence in the manuscript. We also wish to clarify that VOS is not an abbreviation but rather the proper name of the application (a mood and wellbeing tracking app). To avoid confusion, we have now made this explicit in the text at first mention (Page 9), introducing it as "VOS (a mood and wellbeing tracking application)" to make clear that VOS is an app name, not an acronym.

6. **Consistency and Accuracy in Reporting Results. Several instances would benefit from improved consistency and numerical accuracy: • When reporting proportions (e.g., "The majority of apps (n = 85/171)…"), please include the corresponding percentage for consistency. • Avoid repetitive constructions such as "56 (66%, n = 56/85) of the 85…" and streamline reporting while maintaining clarity. • Ensure percentage calculations are accurate and appropriately rounded (e.g., 4/85 is closer to 5% than 4%). • Maintain a consistent reporting format throughout (e.g., (%, n)).**

   Response. We thank the reviewer for this observation. We have addressed each point as follows: (1) the phrase "The majority of apps (n=85/171)" has been revised to include the

corresponding percentage: "(50%, *n* = 85/171)"; (2) the repetitive construction "56 (66%, n=56/85) of the 85 apps" has been streamlined to "56 apps (66%, *n* = 56/85)"; (3) the percentage for 4/85 has been corrected from 4% to 5%; and (4) the reporting format "Three (n=3/85, 4%)" has been standardised to "Three apps (4%, *n* = 3/85)" for consistency throughout. We have reviewed the full manuscript to ensure consistent formatting of the form (%, *n* = *x*/*y*) is applied throughout.

7. **Terminology and Wording Clarifications. • Please clarify whether "free" refers to free to download or free to download and use, where relevant to accessibility. • In the Discussion, please clarify whether the phrase "investigate regular updates" refers to investigating update frequency or to instituting regular updates.**

   Response. We thank the reviewer for raising this comment. The manuscript has been revised to clarify that "free" refers to free to download unless otherwise specified, and that many apps provide only partial free access through freemium models. Regarding the phrase "investigate regular updates" in the Discussion: this was indeed unclear, and we have revised it to "continue releasing regular updates," which more accurately conveys the intended meaning that low user retention reduces developers' incentive to maintain active update schedules.
