# Supplementary material for: Digital mental health apps for young people in Europe: A systematic cross-country analysis
Source: PLOS Digit Health. 2026 Aug 27;5(8):e0001622. doi: 10.1371/journal.pdig.0001622 (PMC13521388; doi:10.1371/journal.pdig.0001622)
Supplement: Supplementary file 3 [file pdig.0001622.s002.docx]

**Table 1**. Output of the initial search per country using the keyword “mental health”.

| **Country** | **App Store** | **Language of the search** | |
| --- | --- | --- | --- |
|  |  | **English** |  |
| Ireland | Apple Store | BetterMe  Breeze  Clarity  Daylio  Headspace  I am - Daily Affirmations  I am Sober  Mental Health Tests  Motivation - Daily quotes  Wysa | N/A |
|  | Google Play | BetterMe  Headspace  Mental: AI Therapy  MindDoc  MindHealth  MyPossibleSelf  Quabble  Voidpet Garden  VOS  Wysa |  |
|  |  | **English** | **French** |
| France | Apple Store | BetterMe  Breeze  Daylio  Finch  I am - Daily Affirmations  I Am Sober  Journal des pensées - TCC  Stoic  VOS  Wysa | Daylio  Fabulous  I am - Daily Affirmations  Lyynk  MindDay: Santé Mentale, Stress  Rootd - Aide pour l'anxiété  Stoic  VOS  WeCare : Santé mentale  Yana : Le compagnon émotionnel |
|  | Google Play | BetterMe  Finch  Headspace  Mental: AI Therapy  MindDoc  MindHealth  Quabble  Voidpet Garden  VOS  Wysa | BetterMe  Clarity  Headspace  MindDoc  Mindspa  Mood Tracker: Self-Care Habits  MindHealth  Remente: Self Care, Wellbeing  Self Therapy: Mental Tests  VOS |
|  |  | **English** | **Portuguese** |
| Portugal | Apple Store | BetterMe  Daylio  Finch  Headspace  Hiwell  Liven: Discover yourself  Motivation - Daily quotes  Stoic  VOS  Wysa | Cingulo  Daylio  Fabulous  Hiwell  I am - Daily Affirmations  InPulse - Heart Rate  Me+ Daily Planner  Motivation - Daily quotes  Stoic  Thera: Mental Health Diary |
|  | Google Play | BetterMe  MindDoc  MindHealth  MyPossibleSelf  Quabble  Stoic  UpLife  Voice: Mental Health Guide  Voidpet Garden  VOS | BetterMe  Cingulo  Hiwell  Mental: AI Therapy  MindDoc  MindHealth  MyPossibleSelf  Quabble  Self Therapy: Mental Tests  VOS |
|  |  | **English** | **Turkish** |
| Turkey | Apple Store | BetterMe  Breeze  Calm  Daylio  Headspace  I am - Daily Affirmations  Me+lifestyle Routine  Meditopia  MindDoc  Wysa | Brainy Train: Zeka Bulmaca  Hiwell  Kişilik Bozuklukları Testi  Mental Sağlık Takipçisi  Mimenta  MoodJourney: Duygu takibi  Stoic  StressEase - Stres İzleyici  Uptime: Kendinizi Geliştirin  Goal Setting - Vision Board 2025 |
|  | Google Play | Betwixt  MindDoc  MindHealth  Self Therapy: Mental Tests  Smiling Mind  SuperBetter: Mental Health  Voice: Mental Health Guide  Voidpet Garden  VOS  Wysa | Heltia: Online Terapi ve Diyet  Hiwell  MindDoc  MindHealth  Mood Tracker ve Günlük Sorular  Relate: Öz Terapi Yolculuğu  Terapi: Psikolojik Testler  Terappin Mental Health Support  UpLife  VOS |
|  |  | **English** | **Croatian** |
| Croatia | Apple Store | Depression Test  Elomia: Mentalna Podrška  Ernest - AI Therapist  Happio  LiftTalk-Coaching & Counseling  Meomind - Therapy Anytime  TalkLife: 24/7 Peer Support  Teen Counseling  Wysa  Youper: AI Mental Health | Belle  BlissLand  Clearway  Dnevnik emocija  FitMindset meditacije i vežbe  Meditacija za smanjenje stresa  Sintelly  Stella  VOS  YouCan |
|  | Google Play | Amaha  Daylio  Headspace  Hedepy  MindHealth  Mind Tracker  Mood Tracker Daily Journal CBT  Remente: Self Care, Wellbeing  UpLife  VOS | Amaha  BetterMe  Headspace  Mind Tracker  Mindspa  My Self Land Emotional Self Help  Don't panic  Smiling Mind  VOS  Youper AI therapy |
|  |  | **English** | **Serbian** |
| Serbia | Apple Store | BetterMe  Calm  Daylio  Finch  Gratitude: Self-care Journal  Headspace  journal & habit tracker - stoic.  MindDoc  VOS  Wysa | FitMindset meditacije i vežbe  No results  No results  No results  No results  No results  No results  No results  No results  No results |
|  | Google Play | BetterMe  Finch  Headspace  Mental: AI Therapy  MindDoc  MindHealth  Quabble  Voidpet Garden  VOS  Wysa | BetterMe  Calm  Harmony my life: Mental Health  I'm Fine: Mental Health Guide  MindHealth  Mindspa  MyPossibleSelf  Self Therapy: Mental Tests  UpLife  VOS |
|  |  | **English** | **Polish** |
| Poland | Apple Store | BetterMe  Breeze  Daylio  Headspace  I am - Daily Affirmations  MindHealth  Quabble  Stoic  VOS  Wysa | DailyBean - simples journal  Daylio  Effecto symptom & mood tracker  Finch  Hapday: AI trener życia  I am - Daily Affirmations  MindHealth  Moodnotes - dziennik nastroju  Don't panic  Stoic |
|  | Google Play | BetterMe  Betwixt  Daylio  Headspace  MindDoc  MindHealth  MyPossibleSelf  Quabble  VoidPet Garden  VOS | BetterMe  Daylio  Hex - ulga w niepokoju  Me: Reflect for Self Awareness  Meditopia  MindHealth  Don't panic  Reminder about taking pills (Przypomnienie o tabletkach)  UpLife  VOS |

**Table 2**. Output of the initial search per country using the keyword “psychological wellbeing”.

| **Country** | **App Store** | **Language of the search** | |
| --- | --- | --- | --- |
|  |  | **English** |  |
| Ireland | Apple Store | Mood Tracker & Emotion Journal  Hiwell  MindHealth  Relate  Boost4u  Tackle Your Feelings  TYF Schools  Memory: My Emotion Jewel Box  Psychology: Happiness  WellSenz | N/A |
|  | Google Play | Remente  Motivation - Daily Quotes  MindHealth  MyPossibleSelf  VOS  Smiling Mind  Aware  Quabble  Betwixt  Wisdom |  |
|  |  | **English** | **French** |
| France | Apple Store | VOS  Hiwell  Envol  Unobravo  Lyynk  MindHealth  Psyhelp  ULY  Motivation - Daily Quotes  Moodnotes | Petit BamBou  Hiwell  VOS  Envol  Unobravo  MindHealth  Lyynk  Motivation - Daily Quotes  Moodnotes  Dr Mood |
|  | Google Play | Remente  ifeel  MindHealth  Betwixt  MyPossibleSellf  VOS  Self Therapy: Mental Tests  Aware  Voice: Mental Health Guide  Smiling Mind | VOS  MindHealth  Self Therapy: Mental Tests  MindDoc  Remente  MindDay  ifeel  Psychology Study & Test Guide  AI Therapist - Voice Chat  Me: Reflect for Self Awareness |
|  |  | **English** | **Portuguese** |
| Portugal | Apple Store | Mood & Emotion Journal  Hiwell  MindHealth  Boost4u  Team24  WellSenz  MError  Charisme  Appreciate - The Journal  The Hapiness Project | Hiwell  HAPDAY IA  Vittude  Yana  Team24  Starbem  AIPsy - your Wellness Ally  NeuroVox  Thrive  Pistolology |
|  | Google Play | Koa Care 360  Mindful Glimpses: Meditation  Mintalitea  Mindberg: Jungian Psychology  Awarefy  Symptom, Mood & Period Tracker  How We Feel  LiveWell  Psychological tests in images  Psicovital | Cingulo  Hiwell  Master's Psychology - School  Yana  BetterMe  Psychological Assessment Test  Self Therapy: Mental Tests  40+ Psychological Tests  MindHealth  MindDoc |
|  |  | **English** | **Turkish** |
| Turkey | Apple Store | Mood Tracker & Emotion Journal  Relate  Hiwell  MindHealth  Got it Life  Therappy  Doer: Overcome Procrastination  WellSenz  Boost4u  MError | MindHealth  Müthiş Psikoloji  No results  No results  No results  No results  No results  No results  No results  No results |
|  | Google Play | MindHealth  Emotions Diary and Mindfulness  Mindary  Sadhguru  Heltia  Relate  Learn Health Psychology  BimbiCalmi  TruMen Eva  PsyVize | Therapy psychological tests  Psychology Guide  Expert Psychology: Online Therapy  Relate  Hiwell  AI Therapist - Voice Chat  Feeling Good  Master's Psychology - School  Psychology - Philosophy Practice  Müthiş Psikoloji |
|  |  | **English** | **Croatian** |
| Croatia | Apple Store | VOS  Mood Tracker and Emotion Journal  Psychology: Happiness  WellSenz  Got it Life  MindHelath  Relate  Mentalport  Boost4u  Youper | Positive Psychology  Awareness  Happio  Psychology: Environment  APA Psychology Dictionary  No results  No results  No results  No results  No results |
|  | Google Play | Mood Tracker Daily Journal CBT  VOS  Therapy psychological tests  Master's Psychology - School  Hiwell  Psychological tests  Mindspa  Psychological issues  ifeel  MindHelath | Hedepy  VOS  Mood Tracker Daily journal CBT  Hiwell  Would you rather? Fun Charades  Guinea Pig Bridge  Platypus: Fairy tales for kids  Helping Hand Mental Health App  Spiky Truth or Dare Game  Amaru |
|  |  | **English** | **Serbian** |
| Serbia | Apple Store | October Health  Hiwell  Boost4u  Winston  Revive & Thrive  Telos  Breathe Magazine  Resilience App  Psychology: Happiness  AltiusLife | No result  No result  No result  No result  No result  No result  No result  No result  No result  No result |
|  | Google Play | Remente  Yana  MindHealth  Psychological Assessment Test  Quabble  ifeel  BetterMe  Aware  Betwixt  Psychological tests | Mindspa  MindHealth  Reflexio  Therapy psychological tests  VOS  AI Therapist - Voice Chat  Depression Test  Master's Psychology - School  Psychological tests  Psychotests, personality tests |
|  |  | **English** | **Polish** |
| Poland | Apple Store | WellSenz  Boost4u  Relate  Evoke  Doer: Overcome Procrastination  Woo  Rapid cyclist  Self help for Trauma  Hiwell  MindHealth | Mindgram  Breathing Exercises: Breazen  Rapid cyclist  Mood Water Tracker: AquaVibe  Aria PA  Timeline - Diary and Notes  Immediate  StartNow+  Psychopathy Test Pro  Nomandful |
|  | Google Play | Remente  MindHealth  Betwixt  VOS  Quabble  MyPossibleSelf  Wysa  UpLife  Sintelly  Goddess | My diary - Journal  Daylio  Moodpress  Daily affirmations mirror  Moodtracker: Self-Care Habits  Hex  CBT Compantion: Therapy app  Hiwell  MindHealth  Awarefy |

**Table 3**. Output of the initial search per country using the keyword “self-help”.

| **Country** | **App Store** | **Language of the search** | |
| --- | --- | --- | --- |
|  |  | **English** |  |
| Ireland | Apple Store | Ahead: Emotions Coach  Clarity: CBT Self Help Journal  Daylio  Finch  Gratitude: Self-Care Journal  Habit Tracker  Headspace  I am - Daily Affirmations  Liven: Discover yourself  MindDoc | N/A |
|  | Google Play | Amaha  BetterMe  Evolve: Self-Care & Meditation  Fabulous Daily Routine Planner  Finch  Gratitude: Self-Care Journal  Mindvalley: Self Improvement  MyPossibleSellf: Mental health  Remente  SELF: Self-Care Affirmations |  |
|  |  | **English** | **French** |
| France | Apple Store | Alux: Self Help & Improvement  Finch  Gratitude: Self-Care Journal  I am - Daily Affirmations  journal & mental health－stoic.  Thought Journal CBT  MINDTRX: Self Growth, SelfHelp  Mindvalley: Self Improvement  Remente  Self Help for Trauma | Alux: Self Help & Improvement  Finch  Gratitude: Self-Care Journal  I am - Daily Affirmations  journal & mental health－stoic.  Journal des pensées - TCC  MINDTRX: Self Growth, SelfHelp  Mindvalley: Self Improvement  Remente  Self Help for Trauma |
|  | Google Play | Amaha  Evolve: Self-Care & Meditation  Fabulous Daily Routine Planner  Finch  Gratitude & Self-Care Journal  Mindvalley: Self Improvement  MyPossibleSellf: Mental health  Remente  SELF: Self-Care and Self-Love  Ultiself \| Self-Improvement | Amaha  Evolve: Self-Care & Meditation  Fabulous Daily Routine Planner  Finch  Gratitude: Self-Care Journal  Mindvalley: Self Improvement  MyPossibleSellf: Mental health  Remente  SELF: Self-Care and Self-Love  Ultiself \| Self-Improvement |
|  |  | **English** | **Portuguese** |
| Portugal | Apple Store | Alux: Self Help & Improvement  Amaha  Betwixt  Catzy: Self-Care Journey  Clarity: CBT Self Help Journal  Finch  Gratitude: Self-Care Journal  journal & mental health－stoic.  Self Help Classics .  Self Help Improvement Quotes! | Bookey: Livros e Autoajuda  BrightMind - CBT TCC Autoajuda  ChatGPT  Dear Anxiety  Mady - Auto-aperfeiçoamento  Minutos de Sabedoria Brasil  Psicologia do Hábito  Rede do Bem  Ultiself \| Self-Improvement  Zen - Meditação e Sono |
|  | Google Play | Diário de humor: bons hábitos  Fabulous: Habit Tracker  Gratitude & Self-Care Journal  Gratitude: Self-Care Journal  Me: Reflect for Self Awareness  Micro Habits: Self-Improvement  MyPossibleSellf: Mental health  Myselfland Emotional Self-Help  Remente  SELF: Self-Care and Self-Love | Daily Affirmations - Positive Me  auto estima e ajuda – otimismo  Autoaperfeiçoament e Bem-Estar  Self-esteem Confidence Booster  Autoestima, el verdadero amor  reflexio  Espelho de afirmações diárias  Psicología de la Autoestima  Self Help Book Summaries  AI Therapist voice talk |
|  |  | **English** | **Turkish** |
| Turkey | Apple Store | Alux: Self Help & Improvement  Clarity: CBT Self Help Journal  Finch  Goddess: Meditasyon Rehberi  Gratitude: Self-Care Journal  HAPDAY-Yaşam Koçu  I am - Daily Affirmations  stoic  Self Help Classics  Self Help Improvement Quotes! | No results  Bluezen - Meditaiton and Sleep  Cogito (MCT)  Mastering emotion  Habit Tracker Master  Psychology: Humanity Weakness  Psychological tests  Selfweller: Self Terapiler  Stop Smoking Pro  We Breathe: Take a Deep Breath |
|  | Google Play | Amaha  BetterMe  MINDTRX: Self Help & Mindset  Mindvalley: Self Improvement  Remente  Self Help for Trauma  Self-help App for the Mind SAM  SELF: Self-Care and Self-Love  Selfweller: Self Terapiler  No results | Hiwell  Kendi Kendine Yardım Kitapları  Kendini Geliştirme Uygulaması  MindHealth  Mindvalley: Self Improvement  Psychological tests  AI Therapist voice talk  No results  No results  No results |
|  |  | **English** | **Croatian** |
| Croatia | Apple Store | Anxiety Solution & Relief  Cleverini  Elomia  Scream Therapy  Awareness  No results  No results  No results  No results  No results | Elomia  Perfect Day: Organizirajte Dan  Sintelly: Terapeutski Chatbot  Svjesnost  VOS  No results  No results  No results  No results  No results |
|  | Google Play | Hiwell  MindHealth  Mood Tracker Daily Journal CBT  Mood Tracker Self Care Habits  Moodi mood tracker and diary  Remente  Therapy psychological tests  SELF: Self-Care and Self-Love  VOS | Aura: Meditation and Sleep CBT  Forest: Focus and Productivity  Headspace  Mood Tracker Daily journal CBT  Moodi Mood tracker and diary  Self Help Book Summaries  Sintelly: Terapeutski Chatbot  VOS  No results  No results |
|  |  | **English** | **Serbian** |
| Serbia | Apple Store | AI Companion - Earkick  Alux: Self Help & Improvement  Amaha  Clarity: CBT Self Help Journal  Finch  Gratitude: Self-Care Journal  MindDoc  Mindvalley: Self Improvement  Self Help Daily- Book Summary  Ultiself Daily Routine Planner | Velora by ieso  No results  No results  No results  No results  No results  No results  No results  No results  No results |
|  | Google Play |  |  |
|  |  | **English** | **Polish** |
| Poland | Apple Store | Alux: Self-Help & Productivity  Amaha  Calm Harm  Clarity: CBT Self Help Journal  Gratitude: Self-Care Journal  journal & mental health－stoic.  Mindvalley: Self Improvement  MindHealth  Utiself \| Self-Improvement  Remente | Habit Tracker Master  MindHealth  Mindvalley: Self Improvement  Psychology: Humanity Weakness  Red Line Life  Remente  Stress test  Self Help Book Summaries  Infinity Loop: Anxiety Relief |
|  | Google Play | Alux: self-help & productivity  Feelway: Self-help with AI  Gratitude: Self-Care Journal  MyselfLand Emotional Self-help  Remente  Self Help Book Summaries  Self Help for Trauma  SELF: Self-Care and Self-Love  Ultiself \| Self-Improvement  MindHealth | Dare: Anxiety & Panic Attacks  Hex  Me: Reflect for Self Awareness  MindSpa  Mindvalley: Self Improvement  Neuronation - trening umysłu  PranaBreath: spokój, medytacja  Self Help Book Summaries  VELORA - Student App  Women’s Guide |

**Table 4**. Output of the initial search per country using the keyword “stress”.

| **Country** | **App Store** | **Language of the search** | |
| --- | --- | --- | --- |
|  |  | **English** |  |
| Ireland | Apple Store | Anti-stress - Relaxing Games  Anti-stress - Relaxing Games  BetterMe  Calm  Feelsy  Headspace  Infinity Loop  Stress Check: Health Monitor  Stress Ball  StressTracker: AI Stress Check | N/A |
|  | Google Play | Antistress - relaxation toys  Breathe: relax & focus  Calm  Energy  Finch  Headspace  Insight Timer  Panic Attack Help - Mind Ease  Simple Habit  Stresscoach |  |
|  |  | **English** | **French** |
| France | Apple Store | Antistress - relaxation toys Apple  Calm  Energy  Heartify  iStress: AI Stress Monitor  StressBall+Relaxation Games  Petit BamBou  Respire: Cardiac Coherence  RespiRelax +  Rootd - Help for Anxiety | Antistress - relaxation toys Apple  Calm  Energy  Heartify  iStress: AI Stress Monitor  StressBall+Relaxation Games  Petit BamBou  Respire: Cardiac Coherence  RespiRelax +  Rootd - Help for Anxiety |
|  | Google Play | Antistress - relaxation toys Google  Balance: Meditation & Sleep  Breathe: relax & focus  Calm  Energy  Headspace  Meditopia  Petit BamBou  Rootd - Help for Anxiety  Stresscoach | Antistress - relaxation toys Google  Balance: Meditation & Sleep  Breathe: relax & focus  Calm  Energy  Headspace  Meditopia  Petit BamBou  Rootd - Help for Anxiety  Stresscoach |
|  |  | **English** | **Portuguese** |
| Portugal | Apple Store | Anti-stress - Relaxing Games  Energy  Feelsy  Hallow  Health Stats  Hearty  Moodpress  Stress monitor for Watch  StressWatch: AI Stress Monitor  Welltory | Antistress - relaxation toys Apple  Hallow  Hearty  IStress: AI Stress Monitor  Moodpress  Stress monitor for Watch  Stressface: HRV Stress Tracker  StressWatch: AI Stress Monitor  Watch Faces App - Watchmaker  Welltory |
|  | Google Play | Antistress - Relaxing Games  Bear Room  Breath Ball  Breathe: relax & focus  Energy  Headspace  Monitor Heart, Stress & Breath  Stress Test  Stresscoach  Trivia Anti-stress - Zen Quiz | Anti-stress - To relax  Breath Ball  Calm  Energy  Forest Island: Relaxing Game  Headspace  Hex  My Oasis  Rootd - Help for Anxiety  Stresscoach |
|  |  | **English** | **Turkish** |
| Turkey | Apple Store | Antistress - relaxation toys Apple  Anti stress top: slime games  Energy  Feelsy  Hearty  Laser: Relaxing & Anti-Stress  Let’s Create! Pottery 2  Pop it Master  StressTracker: AI Stress Check  StressWatch: Al Stress Monitor | Anti-stress Relaxing Games  Anti stress top: slime games  Antistress - relaxation toys Apple  Energy  I am - Daily Affirmations  Pop it Master  Soap Cutting  Moodpress  Stress and Anxiety Relieving Games  Stress Baal |
|  | Google Play | Antistress - relaxation toys Google  Antistress - Relaxation Games  Breath Ball  Energy  My Oasis  Stress Relief: ASMR Relax  Stresscoach  Voidpet Garden  Antistress - Relaxing Games  No results | Energy  Antistress - Relaxation Games  Antistress fidget toys  Anti-stress - To relax  Antistress - Relaxing Games  Hex  Meditopia  Mini Antistress Relaxing Games  My Oasis  No results |
|  |  | **English** | **Croatian** |
| Croatia | Apple Store | Anti-stress - Relaxing Games  Calm  Counselr  Headspace  StressPal: HRV Stress Monitor  No results  No results  No results  No results  No results | Calm  Happify: for Stress & Worry  Moodpress  Stress Monitor for Watch  No results  No results  No results  No results  No results  No results |
|  | Google Play | Antistress - Relaxation Games  Antistress - relaxation toys Google  Antistress - Relaxation Games  Breathe: relax & focus  Calm  Energy  Headspace  Meditopia  My Oasis  No results | Alti  Pop it Master  Antistress - relaxation toys  No results  No results  No results  No results  No results  No results  No results |
|  |  | **English** | **Serbian** |
| Serbia | Apple Store | Anti-stress - Relaxing Games  Energy  Feelsy  Hearty  iStress: AI Stress Monitor  Laser: Relaxing & Anti-Stress  Stress Check: Health Monitor  Moodpress  StressScan - check your stress  StressWatch: AI Stress Monitor | Anti-stress - Relaxing Games  Energy  Feelsy  Hearty  Pillow: Sleep Tracker  Soap Cutting  Stress Baal  Moodpress  Stress Test  StressWatch: AI Stress Monitor |
|  | Google Play | Antistress - relaxation toys Google  Balance: Meditation & Sleep  Breathe: relax & focus  Calm  Energy  Headspace  Hex  Insight Timer  Simple Habit  Stresscoach | Antistress - relaxation toys Google  Breathe: reax & focus  Calm  Energy  Fluid Particle Simulation LWP  Headspace  Hex  Meditopia  Mini Antistress Relaxing Games  My Oasis |
|  |  | **English** | **Polish** |
| Poland | Apple Store | Antistress - relaxation toys Google  Energy  Feelsy  Hearty  Laser: Relaxing & Anti-Stress  Stress Ball  Moodpress  Stress Monitor for Watch  StressWatch: AI Stress Monitor  Welltory | Antistress - relaxation toys Google  Energy  HeartSense  Hearty  Headspace  Pillow: Sleep Tracker  Moodpress  Stress Watch: AI Stress Monitor  VOS  Water Tracker by WaterMinder |
|  | Google Play | Antistress - relaxation toys Google  Trivia Anti-stress - Zen Quiz  Colors: Stress, Sleep, Relax  Feelsy  My Oasis  Smiling Mind  Stress Test  Stressi  Stress test  VOS | Antistress - relaxation toys Google  Trivia Anti-stress - Zen Quiz  Colors: Stress, Sleep, Relax  Feelsy  My Oasis  Smiling Mind  Stress Test  Stressi  Stress test  VOS |

*Note*. Full output of initial app store searches. This table lists all 967 apps identified across all keywords (mental health, psychological wellbeing, self-help, stress), countries (Croatia, France, Ireland, Poland, Portugal, Serbia, Türkiye), platforms (Apple App Store and Google Play Store), and search languages (English and respective national languages).
