# Supplementary material for: Digital mental health apps for young people in Europe: A systematic cross-country analysis
Source: PLOS Digit Health. 2026 Aug 27;5(8):e0001622. doi: 10.1371/journal.pdig.0001622 (PMC13521388; doi:10.1371/journal.pdig.0001622)
Supplement: Supplementary file 4 [file pdig.0001622.s003.docx]

**Table 1**. Cross-country comparison of English-language versus local-language app search outputs.

| **Country** | **English-language search patterns** | **Local-language search patterns** | **Apps explicitly targeting young people identified in searches** | **Dominant app types identified** |
| --- | --- | --- | --- | --- |
| Ireland | English-language searches consistently returned commercially dominant apps, including Headspace, BetterMe, Daylio, MindDoc, Wysa, and Quabble. | N/A | No apps explicitly targeting young people were identified. | Journaling, meditation, mood-tracking, and AI-supported self-help apps dominated search outputs. |
| France | English-language searches returned commercially dominant apps such as BetterMe, Headspace, Wysa, Finch, and Daylio. | French-language searches returned a broader range of partially localised or French-language apps, including Lyynk, WeCare: Santé mentale, MindDay: Santé Mentale and Petit BamBou. | Lyynk and WeCare: Santé mentale explicitly identified young people as their intended audience. | Local-language searches more frequently returned wellbeing, self-help, emotion-tracking, and mindfulness-oriented apps. |
| Portugal | English-language searches returned commercially dominant international apps such as BetterMe, Stoic, Headspace, Daylio, and Wysa. | Portuguese-language searches yielded apps including Cingulo, Vittude, Hiwell, Thera, and HAPDAY IA. | No apps explicitly targeting young people were identified in Portuguese searches. | Searches frequently returned wellbeing, stress-management, and self-help applications. |
| Türkiye | English-language searches returned commercially dominant apps such as BetterMe, Calm, Headspace, Daylio, and Wysa. | Turkish-language searches frequently returned psychology tests, stress-monitoring tools, and general wellbeing applications. Several translated keywords yielded very limited or no results in Apple Store searches. | No apps explicitly targeting young people were identified in Turkish searches. | Stress-management, psychology-test, and self-improvement apps were more common than clinically oriented mental health apps. |
| Croatia | English-language searches returned globally dominant apps including VOS, Youper, Hiwell, and Mood Tracker Daily Journal CBT. | Croatian-language searches returned fewer relevant apps overall, with several searches yielding non-mental-health or unrelated applications. | No apps explicitly targeting young people were identified in Croatian searches. | Local-language searches more frequently returned wellbeing, relaxation, or non-specialised psychology-related applications. |
| Serbia | English-language searches consistently returned commercially dominant apps such as BetterMe, Calm, Headspace, Daylio, and Wysa. | Serbian-language searches yielded very limited results for several keywords, particularly “self-help” and “psychological wellbeing,” with some searches returning no relevant applications. | No apps explicitly targeting young people were identified in Serbian searches. | General wellbeing and stress-related applications were more common than therapeutic or clinically focused apps. |
| Poland | English-language searches returned commercially dominant apps such as BetterMe, Headspace, Daylio, Stoic, and Wysa. | Polish-language searches returned a broader range of mood-tracking, journaling, and wellbeing apps, including Finch, Meditopia, and Hapday | No apps explicitly targeting young people were identified in Polish searches. | Mood-tracking, journaling, mindfulness, and self-help apps were dominant. |

*Note*. This table synthesises patterns observed in the initial app store searches presented in Supplementary Materials 1 and the app descriptions extracted in Supplementary Materials 2. English-language searches consistently returned a broader range of commercially dominant mental health applications across countries, whereas local-language searches frequently yielded fewer results and less evidence of cultural or linguistic adaptation. France demonstrated comparatively greater localisation and local-language app visibility than several other countries, whereas Croatia and Serbia showed the most limited local-language search outputs.
