# Supplementary material for: Digital mental health apps for young people in Europe: A systematic cross-country analysis
Source: PLOS Digit Health. 2026 Aug 27;5(8):e0001622. doi: 10.1371/journal.pdig.0001622 (PMC13521388; doi:10.1371/journal.pdig.0001622)
Supplement: Supplementary file 5 [file pdig.0001622.s004.docx]

**Table 1.** Detailed description of Structured Journaling & Meditation Apps.

| **No** | **Name of the App** | **Overview** | **App Features** | **Language** | **Target Population** | **Cost** |
| --- | --- | --- | --- | --- | --- | --- |
| 1 | Ahead: AI Mental Health | Self-help techniques to help manage emotions and moods. | Science-proven techniques, emotion tracking, goal setting, personalised journey. | English | Adults and children (+4) | Partly Free |
| 2 | Anxiety solution & relief | Self-help tools for stress relief, helping anxiety, depression, and improving mood. | Mindfulness, meditation, breathing tools, sleep stories, fitness routines. | English | Adults and adolescents (+13) | Partly Free |
| 3 | Altius Life | Support tools and access to psychologists and wellbeing experts. | Content, support tools, health goal tracking and professional support. | English | Adults and adolescents (+17)  For organisations staff and their family members under Employee Assistance Programs | Fully Free |
| 4 | Alux: self-help & Improvement | Provides financial and life coaching. | Topics related to personal growth and share a unique perspective, personalised content, journaling. | English | Adults and children (+4) | Partly Free |
| 5 | AriaPA | Self-help and interactive tools for mental health. | Supportive conversations, emotional analysis, journaling, personalised suggestions, relaxation techniques. | Polish | Adults and adolescents (+17) | Fully Free |
| 6 | Aura: Meditation and sleep CBT | Self-help app for better sleep, stress relief and self-improvement. | Meditations, nature and relaxation sounds, life coaching. | English | Adults and children (+4) | Partly Free |
| 7 | Aware: Mindfulness & Wellbeing | Self-help tools for mental health, wellbeing, and inner development. | Science-based exercises, meditations, live guided sessions: peer-to-peer and facilitator-guided. | English | Adults and adolescents (+13) | Fully Free |
| 8 | Balance: Meditation & Sleep | Provides meditations to support mental health, reduce anxiety and stress, improve sleep, and increase focus. | Personalised guided meditations | English | Adults and children (+4) | Partly Free |
| 9 | Bear Room: stress relief | Self-help tools to reduce stress. | Science-based practices like breathing, tapping, humming, affirmation, micro-meditations, therapeutic soundscapes. | English | Adults and children (+4) | Partly Free |
| 10 | Belle PMDD & Period Tracker | Track symptoms related to menstruation while improving mental wellbeing through CBT therapy tools. | Period and cycle tracker, PMDD symptom tracker, CBT Therapy tools, hormonal health reports. | English | Adults and adolescents (+12)  Women | Partly Free |
| 11 | BetterMe: Mental Health | Self-help tools to support mental health. | Meditations, breathing exercises, sleep stories and calming sounds. | English, Czech, French, German, Hungarian, Italian, Japanese, Polish, Portuguese, Romanian, Russian, Simplified Chinese, Spanish, Swedish, Ukrainian | Adults and children (+4) | Partly Free |
| 12 | BlissLand - Simple habit Sleep | Meditation and sound therapy designed to support mental health, reduce stress and improve sleep quality. | Sleep tracker, meditations, sound therapy. | Arabic, Catalan, Croatian, Czech, Danish, Dutch, English, Finnish, French, Greek, Hebrew, Hindi, Indonesian, Japanese, Korean, Malay, Hungarian, Italian, Polish, Portuguese, Romanian, Russian, Slovak, Simplified Chinese, Thai, Traditional Chinese, Turkish, Ukrainian, Vietnamese, Simplified Chinese, Spanish, Swedish | Adults and children (+4) | Partly Free |
| 13 | Breath Ball Stress Relieve | Breathing techniques to help relax, focus, and regain mental balance. | Breathing exercises, calming visuals. | English, French, German, Italian, Spanish | Adults and children (+9) | Fully Free |
| 14 | Breeze: Start Self-Discovery | Self-help tools to discover more about yourself. | Safe space to connect with friends. | English, French, German, Italian, Spanish | Adults and children (+4) | Partly Free |
| 15 | Calm: Sleep & Meditation | Self-help tools to support mental health | Meditation, sleep stories, stretching exercises, relaxing music, daily streaks. | English, French, German, Italian, Japanese, Korean, Polish, Portuguese, Spanish, Traditional Chinese | Adults and children (+4) | Partly Free |
| 16 | Catzy: Self-Care Journey | Self-care tools to support mental health. | Set goals, emotional reflections, mood calendar, focus timer, breathing exercises, sleep helper. | English, Portuguese, Simplified Chinese, Spanish | Adults and children (+4) | Partly Free |
| 17 | CBT Companion: Therapy app | Learn and practice CBT techniques. | Video lessons, exercises to practice CBT-skills, meditations. Clinicians can assign homework, provides health data. | English | Adults and children | Partly Free |
| 18 | Cingulo: Healing and Growth | Self-help tools to promote mental wellbeing. | Emotional profile tests, self-awareness sessions, meditation, techniques for emotional crises, insomnia and sleep quality issues, emotion diary. | English, Portuguese | Adults and adolescents (+12) | Partly Free |
| 19 | Clearway: Sleep, Mood, Breathe | Self-help tools to reduce stress, manage anxiety, improve sleep quality, and cultivate self-awareness. | Emotion diary and analytics, nature sounds, breathing practices. | English, French, German, Italian, Portuguese, Russian, Simplified Chinese, Spanish | Adults and children (+4) | Fully Free |
| 20 | DailyBean - simplest journal | Diary app to record mood flow. | Monthly calendar for mood flow, mood record, statistics that analyze mood and activity. | English, French, Indonesian, Japanese, Korean, Portuguese, Russian, Simplified Chinese, Spanish | Adults and children (+4) | Partly Free |
| 21 | Dare: Anxiety and Panic Attacks | Self-help tools to help overcome anxiety, panic attacks, worry, negative and intrusive thoughts and insomnia. | Meditations, audio guides, mood journal, breathing exercises, support groups, professional support. | English, German, Portuguese | Adults and children (+4) | Partly Free |
| 22 | Daylio Journal - Daily Diary | Self-help tools to support mental, emotional, and physical health. | Self-care bullet journal with goals, statistics. | English, Bulgarian, Czech, Danish, Dutch, Finnish, French, German, Greek, Hindi, Hungarian, Indonesian, Italian, Japanese, Korean, Malay, Norwegian Bokmål, Polish, Portuguese, Romanian, Russian, Serbian, Simplified Chinese, Slovak, Slovenian, Spanish, Swedish, Traditional Chinese, Turkish, Ukrainian | Adults and children (+4) | Partly Free |
| 23 | My Daily Diary - Mood Journal | Diary app to track and capture moods and emotions. | Journaling, mood tracker. | English | Adults and children (+4) | Partly Free |
| 24 | Effecto symptom & mood tracker | Mood, symptom, and health tracker to improve physical and mental health. | Mood and emotions tracker, symptom tracker, sleep tracker, journaling. | English | Adults and children (+4) | Partly Free |
| 25 | Emotions Diary and Mindfulness | Self-help tools to improve psychological well-being, and mental health. | Psychology courses, mental health balance feature, tools for personality and roles including journaling and breathing exercises, personality harmony test. | English | Adults and children | Partly Free |
| 26 | Envol - Meditation & Wellness | Self-help tools to support physical and emotional well-being. | Meditations, 3D sound journeys, breathing exercises, visualization exercises, health habit tracker. | French, English, Spanish | Adults and children (+4) | Partly Free |
| 27 | Evolve: Self-Care & Meditation | Self-help tools to help relax, de-stress and find inner peace. | Meditations, breathing exercises, mindfulness practices, journaling, daily affirmations. | English | Adults and children (+4)  LGBTQIA | Partly Free |
| 28 | Fabulous: Habit Tracker | Self-help tools and habit tracker to improve physical and mental health. | Coaching series, journaling, breathing exercises, daily affirmations, meditation, goal-tracker, coaching series, one-on-one coaching. | English, French, German, Japanese, Portuguese, Simplified Chinese, Spanish | Adults and children (+4) | Partly Free |
| 29 | Finch | Self-care pet including self-care exercises to support mental health. | Self-care exercises, check-ins, mindful exercises, goal tracker, journaling, breathing exercises. | English | Adults and children (+4) | Partly Free |
| 30 | Gratitude: Self-Care Journal | Self-help tools to support mental health. | Journaling, positive affirmations, vision boards, motivation quotes, blog articles, real-life stories, mindful minutes. | English | Adults and children (+4) | Partly Free |
| 31 | Goal Setting - Vision Board 2025 | Self-help tools to keep the mindset strong and focused. | Goal setting, daily affirmations, journaling. | English, Arabic, Danish, Dutch, French, German, Hindi, Indonesian, Italian, Japanese, Korean, Malay, Norwegian Bokmål, Polish, Portuguese, Russian, Simplified Chinese, Spanish, Swedish, Thai, Traditional Chinese, Turkish, Ukrainian, Vietnamese | Adults and children (+4) | Partly Free |
| 32 | Goddess: Self-care & Wellness | Self-help tools to improve mental health and support women dealing with health conditions. | Daily morning rituals and night routines, resources and insights that empower feminine journey, meditation, breathing exercises, yoga, archetype personality analysis, podcasts, daily affirmations. | Turkish, English | Adults and children (+4)  Women | Partly Free |
| 33 | Hallow Prayer & Meditation | Self-help Catholic tools to support mental health. | Christian meditation, daily prayers, sleep bible stories, community. | Portuguese, German, Spanish, French, English, Italian, Polish | Adults and children (+4)  Catholics | Partly Free |
| 34 | Harmony My Life: Mental Health | Self-help tools to promote mental health and wellbeing. | Personalised content, mental health checkup, meditations, articles, videos. | English, German | Adults (+18) | Partly Free |
| 35 | Headspace: Meditation & Health | Self-help tools to manage anxiety, stress, and depression, and promote mental wellness. | Meditations, sleep tools, sleep sounds, sleepcasts, personalised recommendations, exercises. AI-companion, coaching sessions, self-care trackers. | English, French, German, Japanese, Korean, Portuguese, Simplified Chinese, Spanish | Adults and children (+4) | Partly Free |
| 36 | Insight Timer - Meditation App | Self-help tools to reduce anxiety, stress, and improve sleep. | Meditations, music tracks, community features, discussion groups. | English, Dutch, French, German, Italian, Japanese, Portuguese, Russian, Spanish | Adults and adolescents (+13) | Partly Free |
| 37 | Koa Care 360 | Self-help tools to build emotional resilience and address mental health challenges. | Clinically-validated mental wellbeing resources, check-ins, mental health tracker, in-the-moment support. | English | Adults (+18)  Access through employer health plan. | Fully Free |
| 38 | Lyynk | Self-help tools and resources to help young people better understand themselves and measure their wellbeing. It also includes resources to support adults. | Personalised toolbox, set and track goals. | English, French | Young people and their trusted adults. | Partly Free |
| 39 | Mady - Self-Improvement | Self-help tools to achieve goals while supporting mental health. | Goal tracking, habit tracking, mental health check-ins, mindfulness resources. | English, French, German, Portuguese, Spanish | Adults and children (+4) | Partly Free |
| 40 | Mastering emotion | Self-help tools to manage emotions and promote wellbeing. | Tools to help release stress and manage your emotions and readings. | English, Czech, Danish, Dutch, Finnish, French, German, Italian, Japanese, Korean, Polish, Portuguese, Simplified Chinese, Spanish, Swedish, Thai, Traditional Chinese, Turkish, Vietnamese | Adults and children (+4) | Partly Free |
| 41 | Me: Self-reflection for growth | Self-help tools for self-reflection,physical and mental wellbeing. | Check-ins, journaling, personalised courses. | English, German, Spanish | Adults and children (+4) | Partly Free |
| 42 | Me+ Daily Planner | Self-help tools to improve physical and mental health. | Mood-tracker, reminders, build self-care scheduled, goal tracker. | Portuguese, German, Spanish, French, English | Adults and children (+4) | Partly Free |
| 43 | Mental Health Tracker | Self-help tools to track and record mental health symptoms. | Mental health tracker, articles for stress relief and wellbeing, journaling, tools and resources to alleviate anxiety. | English | Adults and adolescents (+16) | Partly Free |
| 44 | MError | Self-help tools to understand emotions and improve self-awareness. | Swipe-based personality, and self discovery tests, connect with trained buddies for support, checklists for self-exploration. | English | Adults and children (+4) | Fully Free |
| 45 | Micro Habits: Self-Improvement | Self-help tools for self-improvement. | Habit tracker, statistics, to-do lists, journaling, streaks. | English | Adults and children (+4) but advise parental guidance | Partly Free |
| 46 | Mind Tracker Journal | Self-help tools for mental health. | Mood tracker, energy tracker, statistics to analyse data, mark events, neurospheres, calendar, smart recommendations. | English | Adults and adolescents (+16) | Partly Free |
| 47 | MindDay: Santé Mentale, Stress | Self-help tools to reduce stress, anxiety, and improve wellbeing. | Videos, structured programs, self-therapy sessions, journaling, mood-tracking, meditation and self-hypnosis. | English, French | Adults and adolescents (+13) | Partly Free |
| 48 | MindDoc: Mental Health Support | Self-help tools to support common mental health challenges such as depression, anxiety, insomnia, and eating disorders. | Mood-tracking, feedback, course library based on CBT. | English, German | Adults and adolescents (+16) | Partly Free |
| 49 | Mindful Glimpses: Meditation | Self-help tools for stress relief. | Meditation and mindfulness, daily reminders. | English | Adults and children (+9) | Partly Free |
| 50 | Mindspa | Self-help tools to support mental health and wellbeing. | Music Therapy, 3D sounds, clarity test, expert tips and tailor content, | English | Adults and children (+4) | Partly Free |
| 51 | Mindvalley: Self Improvement | Self-help tools to improve mindself and self. | Meditation, community, growth tools. | English, French, German, Italian, Portuguese, Russian, Spanish | Adults and adolescents (+13) | Partly Free |
| 52 | Moodi mood tracker and diary | Self-help tools to support mental health. | Journaling, mood tracker, | English, French, German, Portuguese, Russian, Spanish | Adults and children (+4) | Partly Free |
| 53 | Mood Tracker & Emotion Journal | Self-help tools to better understand emotions, track moods, and take care of mental health. | Mood tracker, analyse statistics, emotion calendar. | English, Arabic, French, German, Indonesian, Italian, Japanese, Portuguese, Simplified Chinese, Spanish, Thai, Turkish, Vietnamese | Adults and children (+4) | Partly Free |
| 54 | Mood Journey: Emotions Tracker | Self-help tools to boost mental health and track daily emotions. | Visualization of mood using colors, mood influencers, note-tacking, analytics and statistics, reminders, articles and tips about mental health. | English | Adults and children (+4) | Partly Free |
| 55 | My Diary - Journal | Self-help tools to track moods and write in a journal. | Journaling, mood tracker, visual representation with pixels stat. | English, Czech, Dutch, French, German, Hindi, Italian, Japanese, Polish, Portuguese, Russian, Slovak, Spanish, Swedish | Adults and children (+4) | Partly Free |
| 56 | Myselfland Emotional Self-help | Self-help tools to deal with stress and anxiety. | Relaxation exercises, breathwork, mindfulness, imaginary exercises, audio-guides. | English, Russian | Adults and children (+4) | Partly Free |
| 57 | Nomandful | Basic self-help tools for mental wellbeing. | Mood-tracker, breathing sessions, journaling, relaxing sounds. | English | Adults and adolescents (+13) | Fully Free |
| 58 | Panic Attack Help - Mind Ease | Self-help tools to de-escalate panic attacks quickly and manage anxiety. | Meditation, breathing exercises, journaling, activities, sleep sound, stress management tools. | English | Adults and adolescents (+13) | Fully Free |
| 59 | Petit BamBou: Mindfulness | Self-help tools to support mental health. | Meditations, background music. | French | Adults and children (+4) | Partly Free |
| 60 | Prana Breath: Calm & Meditate | Self-help tools to increase mindfulness and relieve anxiety. | Breathing exercises, progress chart, health tests, sounds. | English | Adults and children | Partly Free |
| 61 | Psyhelp: Therapy & Psychology | Self-help tools to cultivate inner well-being and restore balance in mental health. | Self-therapy plans, meditations, journaling, mood check-in, psychology tests, videos, daily quotes and affirmations, relaxing sounds | English | Adults and children (+4) | Partly Free |
| 62 | Psychology: Happiness | Self-help tools to acquire happiness | Journaling, notes, readings. | English, Arabic, Czech, Danish, Dutch, Finnish, French, German, Italian, Japanese, Korean, Polish, Portuguese, Simplified Chinese, Spanish, Swedish, Thai, Traditional Chinese, Turkish, Vietnamese | Adults and children (+4) | Partly Free |
| 63 | Red Line Life | Self-help tools for self-discovery. | Journaling, meditation, mood-tracker, motivational phrases, tarot as means of self-discovery. | English, Czech, Dutch, Finnish, French, German, Hungarian, Indonesian, Italian, Japanese, Korean, Norwegian, Polish, Portuguese, Romanian, Russian, Simplified Chinese, Slovenian, Spanish, Swedish, Traditional Chinese, Turkish | Adults (+18) | Partly Free |
| 64 | Reflexio | Self-help tools to improve mood and wellness. | Mood tracker, journaling, question diary, reminders. | English | Adults and children | Partly Free |
| 65 | Relate: Self Therapy Journey | Self-help tools for building healthy relationships and improving overall wellbeing. | Mood tracker, daily affirmations, gratitude questions, wellbeing exercises, self-therapy readings and tasks. | English | Adults and adolescents (+16) | Partly Free |
| 66 | Remente: Self Care, Wellbeing | Self-help tools to achieve self-improvement, healthy habits and wellbeing. | Videos, goal setting, day planner, mental health tracker, journaling, expert goal plans, courses and articles. | English | Adults and adolescents (+13) | Partly Free |
| 67 | Rootd: Panic Attacks & Anxiety | Self-help tools for anxiety and panic attacks. | Therapist-approved panic button (CBT techniques), guided deep breathing, anxiety journal, soothing visualizations, stats page, emergency contact, and lessons. | English, Arabic, French, German, Japanese, Polish, Portuguese, Russian, Simplified Chinese, Spanish, Traditional Chinese, Ukrainian | Adults and children (+4) | Partly Free |
| 68 | Sadhguru - Yoga & Meditation | Self-help tools for stress and anxiety | Meditations, daily quotes, videos, articles, podcasts, yoga | English | Adults and adolescents (+16) | Partly Free |
| 69 | Scream Therapy | Provides scream therapy to alleviate anxiety and stress. | Sound detection, calming feedback, scream tracking. | English, Arabic, Catalan, Croatian, Czech, Danish, Dutch, Finnish, German, Greek, Hebrew, Hindi, Hungarian, Indonesian, Italian, Japanese, Korean, Malay, Norwegian, Polish, Portuguese, Romanian, Russian, Simplified Chinese, Slovak, Spanish, Swedish, Thai, Traditional Chinese, Turkish, Ukrainian, Vietnamese | Adults and children (+4) | Partly Free |
| 70 | Self-help App for the Mind SAM | Self-help tools for recording and monitoring wellbeing. | Mood tracker | English | Adults and adolescents (+16) | Partly Free |
| 71 | Sensa - Mental Coach | Self-help tools to support mental health. | Lessons, mood journal, habit-building strategies, weekly assessment, exercises. | English | Adults and adolescents (+13) | Partly Free |
| 72 | Smiling Mind: Mental Wellbeing | Self-help tools for mental fitness. | Meditation and mindfulness, mood tracker, mental fitness tracker. | English | Adults and children (+4) | Partly Free |
| 73 | Stoic: Journal & Mental Health | Self-help tools to improve mental health. | Journaling, meditation, mood tracker, stoic shield to block apps, sleep lessons, quotes and affirmations, therapy notes, daily prompts. | English | Adults and children (+4) | Partly Free |
| 74 | StressEase - Stress Monitor | Self-help tools to reduce stress, improve focus and take care of mental health. | Stress-tracker, insights from health data. | English, French, Portuguese, Spanish, Turkish | Adults and adolescents (+13) | Fully Free |
| 75 | Stresscoach - Anxiety Helper | Self-help tools for anxiety, stress, and panic. | Digital coach, lessons, exercises, mindfulness, relaxation. | English, German | Adults and adolescents (+13) | Partly Free |
| 76 | Stressie | Self-help tools to manage stress and build resilience. | Personalized coping strategies, mindful exercises, yoga. | English | Adults (+18)  Professionals | Fully Free |
| 77 | TalkLife: 24/7 Peer Support | Peer-support community for sharing and connection. | Peer-support community. | English | Adults and adolescents (+16) | Partly Free |
| 78 | Thera AI Mental Health Journal | Self-help tools for anxiety relief. | Journaling, emotion tracker. | English, Dutch, French, German, Italian, Polish, Portuguese, Russian, Spanish, Ukrainian | Adults and children (+4) | Partly Free |
| 79 | ULY: Daily Diary | Self-help tools to support daily life. | Journaling, activity charts, mood tracker. | English, French | Adults and children (+4) | Partly Free |
| 80 | UpLife - Mental Health Therapy | Self-help tools to support emotional balance and personal growth. | AI-based mood tracking, meditations, self-reflection exercises, habit tracker. | English, Ukrainian | Adults and children (+4) | Partly Free |
| 81 | Voidpet Garden: Mental Health | Self-help tools to help with anxiety, depression, and stress. | Journaling, mood tracker, goal setting, meditation and mindfulness, exercises, check-ins, affirmations. | English | Adults and children (+9) | Partly Free |
| 82 | We breathe: take a deep breath | Self-help tools to reduce stress. | Breathing exercises, soothing background videos. | English | Adults and children (+4) | Fully Free |
| 83 | WeCare: Santé mentale | Self-help tools to support mental health. | Emotion-tracker. | French | Young people 18-25 | Fully Free |
| 84 | Wisdom: Learn, Grown, Thrive | Self-help tools to promote mental and physical wellbeing. | Meditations, videos, check-ins, mood tracker, wellbeing plans, sound scapes, yoga. | English | Adults and adolescents (+13) | Fully Free |
| 85 | Zen: Guided Meditation & Sleep | Self-help tools to relieve stress, sleep better, and reduce anxiety. | Meditations, sleep stories, breathing practices, sleep music, yoga. | English, Dutch, French, German, Hindi, Italian, Portuguese, Spanish | Adults and children (+4) | Partly Free |

**Table 2**. Detailed description of Interactive AI-chatbot Apps.

| **No** | **Name of the App** | **Overview** | **App Features** | **Language** | **Target Population** | **Cost** |
| --- | --- | --- | --- | --- | --- | --- |
| 1 | AI Companion – Earkick | AI-Chatbot to help manage anxiety, track mood, build healthy habits, and feel more in control. | AI-chatbot, smart check-ins, progress stats, breathing and CBT exercises, panic button, soundscapes, habit tracker, evidence-based mental fitness strategies. | English, Ukrainian | Adults and children (+4) | Partly Free |
| 2 | AI Therapist – Voice Chat | Self-help tools to support emotional needs. | AI-chatbot, mood and habit tracker, relaxing tools, healing sounds. | English | Adults and children | Partly Free |
| 3 | Amaha: Mental Health self-care | Self-help tools designed for depression, anxiety, and other mental health concerns. | Self-help courses, personalised self-care plan: activities, goal setting and tracking, mood tracking, self-help courses, AI-chatbot, professional support. | English | Adults and adolescents (+16) | Partly Free |
| 4 | Awarefy | AI-powered app to support mental health, including an AI-chatbot and self-help tools. | AI-charts and stats, check-ins, emotion notes, coping lists and routines, audio guides, personal relationship assessments, AI-counselling. | English, Japanese | Adults and adolescents (+13) | Partly Free |
| 5 | Clarity: CBT Self Help Journal | Self-help tools for managing stress, anxiety, and low mood. | AI-chatbot, CBT-techniques, journaling, check-in, mood and emotions tracker | English, French, German, Italian, Japanese, Korean, Portuguese, Simplified Chinese, Spanish, Traditional Chinese | Adults and adolescents (+12) | Partly Free |
| 6 | Counselr: Mental Health AI | AI-chatbot providing mental health support and coaching. | AI-mental health support and coaching. | English | Adults and adolescents (+12) | Partly Free |
| 7 | Elomia: Mental health AI | AI-Chatbot to support emotional and mental wellness. | AI-chatbot, Get tools for mental health self care to improve your wellbeing, reflect on your feelings with supportive reflection exercises, find help for managing problems or emotional overwhelm. | English | Adults and adolescents (+12) | Partly Free |
| 8 | Evoke - zrozum swoje emocje | AI-chatbot providing psychological support. | AI-chatbot, summaries and recommendations, weekly emotional profiles. | Polish | Adults and children | Fully Free |
| 9 | Feelway: AI for Mental Health | Self-help tools and AI-chatbot to reduce dysfunctional feelings. | AI-chatbot, visual representations of emotions. | English | Adults (+18) | Partly Free |
| 10 | Got it Life: AI Mental Health | AI-chatbot to reduce stress, anxiety, and depression. | AI-chatbot, self-talk. | English | Adults and children (+4) | Fully Free |
| 11 | Hapday - AI Life Coach | AI-chatbot provides coaching. | AI-chatbot, coaching, personalised daily plans, meditation, breathing exercises, journaling, mood and habit tracking, sleep stories, goal setting, | English, French, German, Hindi, Indonesian, Italian, Japanese, Korean, Norwegian Bokmål, Polish, Portuguese, Russian, Simplified Chinese, Spanish, Swedish, Turkish, Ukrainian | Adults and adolescents (+17) | Partly Free |
| 12 | Happio: Mental health Toolkit | Self-help tools and AI mental health chatbot. | Mood and emotion tracker, AI-chatbot providing coaching, CBT-based plans. | English | Adults and adolescents (+13) | Partly Free |
| 13 | Liven: Discover yourself | Self-help tools and AI chatbot to understand feelings, discover self-care ideas, and manage overwhelming thoughts. | Set goals, mood tracker, AI-chatbot, wellbeing tests, soundscapes. | English, Arabic, French, German, Italian, Japanese, Korean, Polish, Portuguese, Spanish, Ukrainian | Adults and adolescents (+17) | Partly Free |
| 14 | Meditopia: Sleep, Meditation | Self-help tools to achieve mental resilience and inner peace. | Meditations, bedtime stories, AI-chatbot. | English, Arabic, Azerbaijani, Danish, Dutch, French, German, Italian, Japanese, Korean, Polish, Portuguese, Russian, Spanish, Turkish | Adults and children (+9) | Partly Free |
| 15 | Mental: AI Therapy | Self-help tools for stress and self-improvement | AI-chatbot | English | Adults and children (+9) | Partly Free |
| 16 | Mentalport | AI support to improve mental health | AI-chatbot, mood-tracker, journaling. | English | Adults and children | Partly Free |
| 17 | October Health | Self-help tools for mental fitness. | Live sessions on mental health, AI-companion, mental health assessments, wellbeing tracker. | English | Adults (+18) | Fully Free |
| 18 | Revive & Thrive | Self-help tools to invest in mental wellness proactively. | Live sessions, AI-chatbot, assessments, progress tracker. | English | Adults (+18) | Fully Free |
| 19 | Sintelly - CBT Therapy | Self-help tools and online therapy to enhance mental health and improve overall wellbeing. | AI-chatbot and CBT-techniques. | English | Adults and adolescents (+13) | Partly Free |
| 20 | Stress Ball - Anxiety Relief | Self-help tools for anxiety. | Breathing exercises, journaling, AI-chatbot. | English | Adults and children (+4) | Partly Free |
| 21 | Telos: Life on Purpose | Self-help tools for self-discovery and life design. | AI-chatbot, personalised insights and challenges, tools to build inner skills. | English | Adults and adolescents (+16) | Partly Free |
| 22 | Velora by ieso | Self-help tools to reduce anxiety, stress, and burnout. | AI-chatbot provides content modules, interactive activities, and skill-building exercises. | English, Spanish | Adults and adolescents (+16) | Fully Free |
| 23 | VOS: Mental Health Tracker | Self-help tools to achieve mental peace and stress anxiety relief. | Journaling, personalized plan, breathing exercises, meditation, inspiring quotes and affirmations, AI-chatbot. | English, Czech, French, German, Polish, Portuguese, Russian, Slovak, Spanish, Ukrainian | Adults and children (+4) | Partly Free |
| 24 | Wiston: Daily Companion | Self-help tools to boost self-confidence and reduce feelings of worry, stress or anxiety | AI-chatbot, goal setting, weekly assessments. | English | Adults and adolescents (+13) | Partly Free |
| 25 | Wysa: Mental Wellbeing AI | Self-help tools to support low mood, stress, anxiety, sleep and a whole range of other wellbeing needs. | AI-chatbot meditation, CBT and DBT techniques. | English | Adults and children (+4) | Partly Free |
| 26 | Yana: Your emotional companion | Self-help tools to improve mood or self-esteem, manage anxiety and learn about mental health. | AI-chatbot, mood history, personalised recommendations, exercises. | English, French, Portuguese, Spanish | Adults and adolescents (+13) | Partly Free |
| 27 | Youper: AI Mental Health | Self-help tools to reduce stress, feel calmer, boost mood, and build healthier relationships. | AI-chatbot. | English | Adults and children (+9) | Partly Free |
| 28 | Woo | Self-help tools for stress, relationships, emotions, and everyday challenges | AI-chatbot | English | Adults (+18) | Fully Free |

**Table 3.** Detailed description of apps that provide a link to online therapists.

| **No** | **Name of the App** | **Overview** | **Type of therapists** | **Language** | **Target Population** | **Cost** |
| --- | --- | --- | --- | --- | --- | --- |
| 1 | Müthiş Psikoloji | Guide individuals in their mental health, personal development, and communication. | Clinical psychologist to provide mental health support. | Turkish | Adults and children (+4) | Fully Free |
| 2 | Hedepy | Psychotherapy to promote mental wellbeing. | Certified psychologist. | English | Adults (+18) | Fully Free |
| 3 | Helping Hand Mental Health App | Mental health support for low mood, anxiety, stress, depression, and relationship crises and difficulties. | Online psychotherapists. | English | Adults (+18) | Fully Free |
| 4 | Heltia: Online Therapy and Diet | Online consultations on mental health, nutrition, and physical wellness. | Clinical psychologist or psychological counselors. | English | Adults and adolescents (+16) | Fully Free |
| 5 | Hiwell Therapy & Mental Health | Online psychological support | Clinical psychologists and psychiatrists. | English, Azerbaijani, Catalan, French, Greek, Italian, Portuguese, Romanian, Spanish, Turkish | Adults and children (+4) | Fully Free |
| 6 | ifeel | Professional support as well as courses to reduce stress and deal with anxiety. | Clinical psychologists. | English, French, German, Hebrew, Italian, Portuguese, Spanish | Adults and adolescents (+16) | Fully Free |
| 7 | LiftTalk-Coaching & Counseling | Space of guidance and support to find emotional stability, mental clarity, and relationship growth. | Certified professionals. | English, Arabic | Adults (+18) | Fully Free |
| 8 | Mindgram | Support and guidance for mental health and wellbeing. | Certified specialists via anonymous chat or live online sessions. | English | Adults and adolescents (+16) | Fully Free |
| 9 | Psicovital | Online psychologist platform providing psychological care. | List of psychologists, schedules, online chat, video calls, and an emergency line. | English | Adults and adolescents (+16) | Fully Free |
| 10 | Starbem Online Therapy | Connects to therapists to facilitate access to mental health support. | Therapists. | English | Adults and adolescents (+16) | Fully Free |
| 11 | Team24 | Access psychological support. | Qualified and certified psychologists. | English | Adults and children (+4)  Exclusively for the employees of partner companies | Fully Free |
| 12 | Teen Counseling | Connects to therapists who specialise in helping teens. | Accredited, trained, and experienced licensed U.S. psychologist (PhD / PsyD), licensed marriage and family therapist (LMFT), licensed clinical social worker (LCSW), licensed professional counselor (LPC), or similar applicable recognized professional certification. | Dutch, French, German | Adults and adolescents (+13)  Teens | Fully Free |
| 13 | Terappin Mental Health Support | Mental health support from therapists online. | Clinical psychologists. | English | Adults and children (+4) | Partly Free |
| 14 | TruMen Eva | Connects to group chats and one-on-one conversations providing psychological support. | Choose an online psychologist or a virtual psychologist. | English | Adults and children | Fully Free |
| 15 | Unobravo | Schedule online appointments to support mental health. | Psychologists via videoconferencing or chat. | French, English, Spanish, Italian | Adults and adolescents (+16) | Fully Free |
| 16 | Vittude | Connects to psychological support. | Qualified psychologists. | English | Adults and children | Partly Free |

**Table 4.** Detailed description of content-focused Apps.

| **No** | **Name of the App** | **Overview** | **Language** | **Target Population** | **Cost** |
| --- | --- | --- | --- | --- | --- |
| 1 | Daily Affirmations - Positive Me | Daily quotes and positive affirmations. | English, Arabic, Dutch, French, German, Italian, Japanese, Korean, Portuguese, Russian, Simplified Chinese, Spanish, Swedish, Thai, Traditional Chinese, Turkish | Adults and children (+4) | Partly Free |
| 2 | Auto estima e ajuda – otimismo | Lessons about self-esteem and the impact of negative emotions. | Portuguese | Adults and children | Partly Free |
| 3 | Autoaperfeiçoament e Bem-Estar | Blogging, self-improvement books, self-love quotes. | Portuguese | Adults and children but parental supervision advised | Partly Free |
| 4 | Self-esteem Confidence Booster | Self-care chapters and daily affirmations on self-esteem, self-acceptance, self-compassion, self-respect and self-judgement. | English | Adults (+18) | Partly Free |
| 5 | Daily affirmations Mirro | Daily affirmations, music. | English, Croatian, Czech, Danish, Dutch, Finnish, French, German, Greek, Hungarian, Indonesian, Italian, Japanese, Korean, Norwegian Bokmål, Polish, Portuguese, Romanian, Russian, Simplified Chinese, Slovak, Spanish, Swedish, Turkish, Ukrainian | Adults and children (+4) | Partly Free |
| 6 | How We Feel | Step-by-step videos to address negative thoughts, promote cognitive strategies and emotional wellbeing. | English | Adults and children (+4) | Fully Free |
| 7 | I am - Daily Affirmations | Daily affirmations to build self-esteem and change negative thought patterns. | English, Arabic, Dutch, French, German, Italian, Japanese, Korean, Portuguese, Russian, Simplified Chinese, Spanish, Traditional Chinese, Turkish | Adults and children (+9) | Partly Free |
| 8 | Lumiere: Ease Stress & Anxiety | Gratitude and acceptance practices to develop psychological flexibility. | English | Adults and children (+4) | Partly Free |
| 9 | Meomind - Therapy Anytime | Library of recorded therapy sessions. | English | Adults and children (+4) | Partly Free |
| 10 | MINDTRX: Self Growth, Self Help | Audios to promote self-development, personal growth, self-transformation, and overall wellbeing. | English | Adults and adolescents (+16) | Partly Free |
| 11 | Minutos de Sabedoria Brasil | Self-help phrases. | English | Adults and children | Partly Free |
| 12 | Motivation - Daily quotes | Motivational reminders and quotes on self-esteem, relationships, and dealing with stress. | English, Arabic, Dutch, French, German, Italian, Japanese, Korean, Portuguese, Russian, Simplified Chinese, Spanish, Traditional Chinese, Turkish | Adults and children (+9) | Partly Free |
| 13 | Pistolology | Library of lessons and podcasts designed to promote self-awareness, enhance cognitive skills, and foster positive behavioral changes | English, Portuguese | Adults and children (+4) | Fully Free |
| 14 | Psicología de la Autoestima | Reading chapters to learn about self-esteem. | Spanish | Adults and adolescents (+12) | Fully Free |
| 15 | Psychology: Humanity Weakness | Reading chapters to understand weaknesses and provide strategies for turning them into strengths. | English, Arabic, Czech, Danish, Dutch, Finnish, French, German, Italian, Japanese, Korean, Polish, Portuguese, Simplified Chinese, Spanish, Swedish, Thai, Traditional Chinese, Turkish, Vietnamese | Adults and adolescents (+13) | Partly Free |
| 16 | Self Help Book Summaries | Short book summary feature for the best self-help for anxiety | English | Adults and adolescents (+12) | Partly Free |
| 17 | Self Help Daily- Book Summary | Self-help books and audios on anxiety management, relationship building, productivity, health and wellness. | English, Arabic, Bengali, Bulgarian, Catalan, Croatian, Czech, Danish, Dutch, Finnish, French, Georgian, German, Greek, Hebrew, Hindi, Hungarian, Indonesian, Italian, Japanese, Korean, Lithuanian, Malay, Malayalam, Marathi, Persian, Polish, Portuguese, Romanian, Russian, Simplified Chinese, Spanish, Swedish, Tamil, Thai, Turkish, Vietnamese | Adults and children (+4)  Professionals | Partly Free |
| 18 | SELF: Self-Care Affirmations | Daily affirmations to focus on growth, increase self-esteem, and build healthy patterns of self-love. | English | Adults and children (+4) | Partly Free |
| 19 | Voice mental health guide | Audio-guides on “hypnotelling” to support mental health. | English | Adults and adolescents (+13) | Partly Free |
| 20 | YouCan | Daily affirmations. | English, Russian, Spanish | Adults and children (+4) | Partly Free |

**Table 5.** Detailed description of gamified apps.

| **No** | **Name of the App** | **Overview** | **Language** | **Target Population** | **Cost** |
| --- | --- | --- | --- | --- | --- |
| 1 | Anti stress top: slime games | Relieve tension by squeezing the anti-stress ball, jelly ball or realistic slime on the screen. | English, Russian | Adults and children (+4) | Partly Free |
| 2 | Antistress - Relaxation Games | Play with a fidget spinner, smash some phones to relieve stress, pop bubbles with bubble wrap to release stress. | English | Adults and children (+4) | Partly Free |
| 3 | Antistress - relaxation toys Apple | Collection of antistress toys. | English | Adults and children (+4) | Partly Free |
| 4 | Antistress - relaxation toys | Collection of toys for relaxation, diversion, or a moment of distraction. | English | Adults and children (+4) | Partly Free |
| 5 | Antistress fidget toys | Relaxing games, each designed to provide soothing and satisfying experiences for anxiety relief, stress relief, or simply passing the time with calming games. | English | Adults and children (+4) | Partly Free |
| 6 | Betwixt - The Mental Health Game | Fantasy text-based game to improve mental health, relieve stress and anxiety, and find clarity, confidence and calm. | English | Adults (+18) | Partly Free |
| 7 | Energy: Anti-Stress Loops | Calming and anxiety-relieving puzzle game. | English, French, German, Japanese, Korean, Simplified Chinese, Spanish, Traditional Chinese | Adults and adolescents (+13) | Partly Free |
| 8 | Feelsy: Anxiety Relief | 3D/4D textures that will reduce stress, anxiety and help with sleep. | English, Arabic, French, German, Japanese, Korean, Portuguese, Simplified Chinese, Spanish | Adults and children (+4) | Partly Free |
| 9 | Fluid Particle Simulation LWP | Create satisfying digital art and relieve stress. | English | Adults and children | Partly Free |
| 10 | Forest Island: Relaxing Game | Relax and unwind with the animals, and experience stress relief and happiness by growing various natural habitats. | English, French, German, Indonesian, Italian, Japanese, Korean, Portuguese, Russian, Simplified Chinese, Spanish, Thai, Traditional Chinese, Vietnamese | Adults and adolescents (+13) | Partly Free |
| 11 | Happify: For Stress & Worry | Science-based activities and games to overcome negative thoughts, stress, and life's challenges.. | English, French, German, Italian, Japanese, Portuguese, Simplified Chinese, Spanish | Adults and children (+4) | Fully Free |
| 12 | Hex - Anxiety relief | Build loops and relieve stress. | English, French, German, Japanese, Korean, Simplified Chinese, Spanish, Traditional Chinese | Adults and children (+4) | Partly Free |
| 13 | Infinity Loop: Anxiety Relief | Guide a ball as it smoothly orbits an infinity-shaped path to relax. | English | Adults and children | Partly free |
| 14 | Laser: Relaxing & Anti-Stress | Connect your energy sources to batteries to recharge and relieve stress. | English, Arabic, Danish, Dutch, Finnish, French, German, Indonesian, Hindi, Italian, Japanese, Korean, Portuguese, Russian, Simplified Chinese, Spanish, Turkish, Thai, Vietnamese, Traditional Chinese | Adults and children (+4) | Partly Free |
| 15 | Let 's create! Pottery 2 | Experience calmness, tranquility, and enhance your emotional and mental well-being by making art. | English, French, German, Italian, Japanese, Korean, Polish, Portuguese, Russian, Spanish | Adults and children (+4) | Partly Free |
| 16 | Mini Anti Stress Relaxing Games | Collection of relaxing antistress games. | English | Adults and children | Partly free |
| 17 | My Oasis: Relaxing, Satisfying | Zen environment and calming atmosphere to relieve stress. | English, French, German, Indonesian, Italian, Japanese, Korean, Russian, Simplified Chinese, Spanish, Thai, Traditional Chinese, Turkish, Vietnamese | Adults and children (+4) | Partly Free |
| 18 | Norbu: Calming & Relaxation | Anti-stress app to manage stress and emotions on the physical level using mindfulness in a gamified way. | English, Arabic, French, German, Hindi, Italian, Japanese, Korean, Persian, Portuguese, Russian, Simplified Chinese, Spanish, Turkish, Vietnamese | Adults and children | Partly Free |
| 19 | Pop it Master: asmr calm games | Press all the bubbles to open the next colorful toy and relieve stress. | English, Arabic, French, German, Hindi, Indonesian, Italian, Japanese, Korean, Portuguese, Russian, Simplified Chinese, Spanish, Turkish, Vietnamese | Adults and children (+4) | Partly Free |
| 20 | Quabble: Daily Mental Health | Mental wellness routines and mental health games. | English | Adults and adolescents (+13) | Partly Free |
| 21 | StressBall+Relaxation Games | Anti-stress games and playful meditations, as well as breathing techniques for relaxation. | French, German, English, Danish, Spanish, Finnish, Hungarian, Italian, Norwegian, Dutch, Polish, Portuguese, Russian, Swedish, Czech, Turkish, Ukrainian | Adults and children (+4) | Partly Free |
| 22 | Trivia Anti-stress - Zen Quiz | Quizzes to provide stress relief and relaxation. | English, Czech, Danish, Dutch, Finnish, French, German, Greek, Hungarian, Italian, Japanese, Korean, Norwegian Nynorsk, Polish, Portuguese, Romanian, Russian, Spanish, Swedish, Turkish, Ukrainian | Adults and children (+4) | Partly Free |

*Note*. Data extraction tables for all 171 included apps. All data were obtained directly from publicly available app store listings accessed between April 28 and May 5, 2025. No data was obtained through correspondence with developers or from external sources outside the app store listings.
