# Supplementary material for: Digital mental health apps for young people in Europe: A systematic cross-country analysis
Source: PLOS Digit Health. 2026 Aug 27;5(8):e0001622. doi: 10.1371/journal.pdig.0001622 (PMC13521388; doi:10.1371/journal.pdig.0001622)
Supplement: Supplementary file 6 [file pdig.0001622.s007.docx]

**Response to reviewers**

We thank the editor and reviewers sincerely for their constructive response to our revised manuscript and their recommendation about how the manuscript could be further improved. We have carefully addressed all comments and provided detailed point-by-point responses below, including the corresponding page and line numbers where revisions have been made in the updated manuscript. For clarity, we have also summarized the changes within our responses.

**Reviewer #2:**

1. **Format: n/N (%) OR % (n/N). Example: 312/699 (44.6%) of participants had viral suppression or Viral suppression was observed in 312/699 participants (44.6%). There was an error in the interpretation of my comment. There is still some repetition e.g. Regarding language availability, nine (41%, n=9/22) apps were offered only in English (NOTE nine appears twice. THIS IS BEST PRESENTED AS 41% (9/22); one (5%, n=1/22) - THIS CAN BE PRESENTED AS 5%(1/22). Hope this is clear. YOU CAN CONFIRM WITH PLOS DH FORMATTING GUIDELINES OR PREVIOUS PUBLISHED ARTICLES.ALL THE BEST.**

**Response.** We thank the reviewer for this clarification. We have now thoroughly revised the Results section to ensure consistent use of the format % (n/N) throughout, eliminating all instances of redundant repetition (e.g., “nine [41%, n=9/22]” has been corrected to “41% (9/22)”). The word-number spelling of counts has been removed where it preceded the bracketed statistic. This formatting revision has been applied consistently across all app category subsections.

For example, on Page 15, Line 2-12, the Results section now reads:

“Peer-support community access was available in 1% (1/85) of apps, while another 1% (1/85) claimed to provide scream therapy for stress and anxiety relief. Cognitive Behavioural Therapy (CBT) techniques were incorporated in 4% (3/85) of apps, and 19% (16/85) included sleep-related tools, such as sleep music, sleep stories, sleep trackers, or sleep sounds. Financial and life coaching features were identified in 1% (1/85) of apps, whereas 4% (3/85) included self-discovery tools.

Most journaling and meditation apps were available in a single language (52%, 44/85). Among these, 93% (41/44) were available exclusively in English, 5% (2/44) in French, and 2% (1/44) in Polish. Apps supporting two languages accounted for 12% (10/85), most commonly combining English with French or German. The remaining 36% (31/85) supported more than two languages, ranging from three to thirty-two languages.”

**Reviewer #3:**

1. **The authors, as part of a European research project, examined the availability of mental health apps in their respective countries, focusing on the target group of “young users.”
   I was not involved in the review of the first version of the manuscript. However, I have read the previous review reports and the authors’ response, and I have contributed to assessing whether the feedback was adequately addressed. I appreciate the extended discussion, but I would also have liked that the results were presented in a different form, to better prepare for the discussion and aligning better with the research aim. I recommend looking into the following aspects of the paper, which in my opinion have not been tackled satisfactorily:**

**Target group “young people”. The authors state that the study focuses on young people (from children to adolescents). The authors indicate that their analysis is based solely on information in the App Store/Play Store: We acknowledge that app store age ratings (4+, 12+, 18+) reflect platform content guidelines rather than intended target populations, and that these do not map straightforwardly onto our conceptualisation of young people (...). Therefore, age-related classifications reported in this study reflect app store metadata rather than verified target populations. In other words, the way the app actually looks and functions has not been evaluated. I therefore do not find it convincing when the authors claim to have assessed apps specifically for young people with mental health problems. I would rather distinguish between apps that do not specify a clear target group and apps that do; one of the results could then be the number of apps that explicitly target young people, this would make the “target population” column in Table 1 (appendix) and table 3 (manuscript) more meaningful. An age rating such as “4+” does not necessarily mean that an app is intended for use by children/teenagers or has been developed with this group in mind. Are the language, look and feel, and the type of interventions tailored to this target group? Probably not. However, I consider the research team sufficiently qualified to also make a substantive judgement about the suitability of these “4+apps” for young people.**

**Response**. We sincerely thank the reviewer for their constructive feedback, and we really appreciate the reviewer’s detailed engagement with the manuscript. In response, we have substantially revised the manuscript to strengthen the analytical dimensions of the Results section, clarify the conceptualisation of “young people,” and more explicitly present cross-country and cross-language differences.

We fully agree that the age rating system used by app stores (4+, 12+, 18+) does not map directly onto whether an app has been designed for or is suitable for young people. Our original intention was to examine the digital mental health apps most likely to be accessible and visible to young people within app stores, rather than to imply that all included apps were explicitly youth-targeted. While this limitation was acknowledged in the previous version of the manuscript, we agree that it was not sufficiently integrated throughout the Results and Discussion sections.

In response, we revised the manuscript throughout to distinguish between: (1) apps explicitly targeting young people or youth-specific populations in their descriptions; and (2) apps broadly accessible to young people based on app store classifications.

We also revised wording throughout the manuscript to avoid overstating that included apps were specifically “for young people.” For example, the Introduction now states (Page 12, Line 9-13):

“The present study addresses these gaps by conducting a cross-country and cross-language comparison of mental health apps accessible to young people across seven European countries.”

Additionally, we clarified in the Methods section that app store age ratings reflect platform metadata rather than verified target populations or developmental suitability. On Page 6, Line 20-24, the manuscript now states:

“Importantly, this study did not assume that apps categorised as “4+” or “12+” were specifically designed for children or adolescents. Rather, these classifications were used to examine which apps are broadly accessible and visible to young people within commercial app stores. Where app descriptions explicitly identified a target population (e.g., young adults, or families), this information was extracted separately.”

We also revised Table 3 to replace “Target user” terminology with “App store age classification and stated target populations,” thereby more accurately reflecting the nature of the extracted app store data.

Additionally, in the Results section, we added a dedicated paragraph highlighting the relatively small number of apps that explicitly identified young people as their intended audience. Based on the app store descriptions presented in Supplementary Materials 2, we identified only a limited subset of apps explicitly targeting young people, including Lyynk and WeCare: Santé mentale. This finding has now been highlighted as an important result because the vast majority of apps did not specify any explicit youth-oriented design intent. The manuscript now states (Page 11, Line 22-24; Page 12, Line 1-8):

“It is important to note that across all 171 included apps, only a small subset explicitly named young people as their primary intended audience in the app store description. Apps that explicitly targeted young people included Lyynk (described as designed to help young people better understand themselves, alongside resources for trusted adults), WeCare: Santé mentale (designed for young people aged 18–25 years), and one structured journaling and meditation app specifically designed for young adults and their trusted adults. The vast majority of apps did not specify any youth-focused design intent, which is itself a notable finding given this study’s focus on young people’s digital mental health access. App store age ratings (4+, 12+, 17+/18+) reflect content thresholds applied by platform operators rather than developer-stated target populations, and should not be interpreted as indicators of youth-appropriate design. .”

Although the present study did not include a formal qualitative evaluation of app design or developmental appropriateness, we reviewed app descriptions more interpretively during manuscript revision to identify whether developers explicitly referenced young people, adolescents, students, families, or youth-focused support within app store materials. This additional distinction has now been incorporated throughout the Results and Discussion sections. The Discussion section now states (Page 26, Line 5-13):

“Additionally, although this study examined which apps were accessible and visible to young people within commercial app stores, the research team did not conduct a qualitative assessment of app interface design, developmental appropriateness, readability, visual presentation, or intervention suitability for youth populations. As such, the findings should not be interpreted as evidence that apps categorised as ‘4+’ or ‘12+’ were specifically designed for or appropriate for children or adolescents. Future research should incorporate direct app testing and qualitative evaluation methods to assess the extent to which digital mental health applications are genuinely youth-centred in their design, content, and therapeutic approach.”

2. **Tables, visualisations, and cross-country differences. The tables in the appendix are currently purely descriptive. I recommend using the tables more actively in the Results section and, for example, adding other types of visualisation and colour coding to illustrate differences between what an English-language search versus a native-language search yields in terms of apps. For example, could the contrast between apps in English and in the local language be developed further? For example, by explicitly comparing availability, types of interventions, or target groups per language category. One of the aims was to identify cross-country differences. Because the data are not presented by country in the Results, I feel that these cross-country differences have not yet been sufficiently elaborated, although the Discussion points at some interesting findings. For example, the authors state: This pattern underscores the global dominance of English-language apps and suggests limited localisation or cultural adaptation. This conclusion would be stronger if cross-country patterns were more clearly shown in the results, e.g. in a graph or other visual. Table 2 (Manuscript) The main point the authors seek to make with Table 2 in the Results section is not clear to me in terms of the article’s central message, because it is about one specific app.**

**Response.** We thank the reviewer for this valuable observation and agree that the previous version of the manuscript did not sufficiently synthesise cross-country and cross-language patterns within the Results section.

In response, we have added a new subsection entitled “Cross-Language and Cross-Country Differences” within the Results section. This subsection explicitly compares English-language and local-language search outputs across countries and synthesises broader cross-country patterns rather than presenting findings only descriptively by app category. Specifically, we now highlight that English-language searches consistently yielded a broader range of commercially dominant apps across all countries; Serbian and Croatian local-language searches returned minimal or no results for several keywords; French- and Portuguese-language searches yielded comparatively more localised and linguistically adapted apps than other countries; local-language searches more commonly returned general wellbeing and stress-management apps rather than therapeutic or clinically oriented mental health applications. The Results section now states (Page 10, Line 1-14; Page 11, Line 1-12):

“Cross-country differences were most evident in the contrast between English-language and native-language searches. English-language searches consistently returned a broader range of commercially dominant apps across all countries, including Headspace, Wysa, Daylio, MindDoc, and BetterMe. In contrast, searches conducted in local languages frequently yielded fewer results, particularly in Croatia and Serbia, where some translated keywords returned minimal or no relevant applications. For example, Serbian-language searches using terms related to “psychological wellbeing” returned no Apple App Store results for several keywords, whereas Croatian-language searches often returned only a small number of locally relevant applications.

Differences were also observed between countries in the extent of localisation and cultural adaptation. French- and Portuguese-language searches generally returned a broader range of locally adapted or partially localised apps, including apps available in French or Portuguese interfaces, whereas searches conducted in Serbian, Croatian, and Turkish more commonly returned English-language commercial apps with limited localisation beyond translation of the app title or description.

Native-language searches were also more likely to return apps focused on general stress reduction, self-help, or wellbeing rather than apps describing therapeutic or clinically oriented support. Across countries, relatively few apps appeared to be specifically tailored to local cultural or linguistic contexts. Most apps identified through local-language searches were either direct translations of English-language commercial apps or applications with limited localisation beyond interface language.

Overall, these findings suggest that young people searching in English may encounter a broader and more diverse range of digital mental health resources than users relying solely on native-language search terms. A country-level comparison of English-language versus local-language app search outputs is presented in Supplementary Materials 2.”

We also revised the introductory text surrounding Table 2 to clarify its analytical relevance to the manuscript’s central argument. Specifically, Table 2 is now explicitly framed as an illustration of how app store metadata and app “popularity” metrics vary substantially across national app store contexts, even for the same globally dominant application. The revised Results section now states (Page 9, Line 13-19):

“Cross-country discrepancies were also observed in app store metadata. These differences suggest that app visibility and user engagement metrics may vary across national app store contexts, even for globally dominant applications, showing that even the same app is presented differently to users depending on which country’s store they access. For example, Headspace, the most globally visible app in our sample, showed notable variation in user ratings and review counts across country-specific Apple Store listings (Table 2), indicating that app “popularity” as indexed by ratings is not directly comparable across national contexts.”

3. **Table 3 (manuscript): • I would reconsider the age-based categorisation, for the reasons outlined under point 1 above. • Is it relevant for this article that some international apps are also available in Japanese, Arabic, etc.? It seems to me that three categories would be sufficient: “English only,” “local language only” (from the countries in this study), and “several languages.” • Is it possible to retrieve information about where the apps originate from—that is, in which country they were developed? This would add a useful dimension to the analysis. • Is it not somewhat surprising that apps providing access to therapy are free of charge, given that this appears to be the most resource-intensive form of care? Presumably, users have to pay for the consultation itself, or the app is only available to people who already have a care contract. This is an important nuance to highlight when discussing this table and in the Discussion.**

**Response.** We thank the reviewer for this important observation. As noted in our response to Comment 1, the age-based rows in Table 3 have been relabelled to reflect app store classifications rather than verified developmental suitability, and stated target populations rather than developmental targeting. We believe retaining these variables, while clearly acknowledging their limitations, provides useful contextual information regarding platform content thresholds and accessibility. In response to the reviewers recommendation regarding language availability, we substantially revised the structure of Table 3. The previous language availability rows have been collapsed into four meaningful categories: English only, local language only, English + local language, and multilingual (>2 languages).

We agree that app developer country of origin would provide an important additional dimension to the analysis. However, developer location information was inconsistently available across app stores and was not systematically extracted during data collection. Consequently, we were unable to reliably incorporate this variable into the present study. We have now acknowledged this as a limitation and direction for future research within the Discussion section. See Page 27, Line 23-25; Page 28, Line 1-6:

“App developer country of origin was not systematically extracted because this information was inconsistently available across app store platforms and developer profiles. Consequently, the present study was unable to assess whether apps were developed locally or internationally, or the extent to which app origin may have influenced linguistic adaptation, cultural relevance, or localisation practices. Future research should examine developer origin and regional development patterns in greater detail to better understand how digital mental health resources are distributed and adapted across different cultural and linguistic contexts.”

Finally, we thank the reviewer for highlighting the important distinction between “free download” and genuinely free access to professional therapy services. In response, we added the following clarification to the Discussion section (Page 24, Line 2-7):

“Notably, although most apps connecting users to online therapists were technically free to download, this did not necessarily imply free access to therapy itself. In many cases, app descriptions indicated that consultations, subscriptions, employer-based healthcare access, or session fees were required to access professional services. Therefore, “free download” classifications should not be interpreted as equivalent to free mental health care access.”

4. **All in all I feel the paper has improved, but the results section is too descriptive.**

**Response**. We sincerely thank the reviewer for this thoughtful observation and agree that the previous version of the manuscript relied heavily on descriptive reporting. In response, we have revised the Results section to strengthen the analytical and comparative dimensions of the manuscript and to align more closely with the study aims regarding cross-country and cross-language accessibility.

Specifically, we incorporated interpretive synthesis throughout the Results section to move beyond descriptive categories of apps, and instead highlight broader patterns relating to localisation, accessibility, and digital inequality. We added a new subsection entitled “Cross-Language and Cross-Country Differences”, which synthesises cross-country trends and explicitly compared English-language and local-language app visibility across countries.

Supplementary Table S2 now provides a cross-country comparison of English-language versus local-language app search outputs. We believe these revisions substantially strengthen the analytical depth of the Results section.
