# Supplementary material for: Digital mental health apps for young people in Europe: A systematic cross-country analysis
Source: PLOS Digit Health. 2026 Aug 27;5(8):e0001622. doi: 10.1371/journal.pdig.0001622 (PMC13521388; doi:10.1371/journal.pdig.0001622)
Supplement: Peer review history

# Peer review: Decision Letter 0

**DOI:** 10.1371/journal.pdig.0001622.r001

**Authors:** Mengyu Wang, Girum Zewude

16 Mar 2026

PDIG-D-25-01183

Digital Mental Health Apps for Young People in Europe: A Systematic Cross-Country Analysis

PLOS Digital Health

Dear Dr. Madrid Cagigal,

Thank you for submitting your manuscript to PLOS Digital Health. After careful consideration, we feel that it has merit but does not fully meet PLOS Digital Health's publication criteria as it currently stands. Therefore, we invite you to submit a revised version of the manuscript that addresses the points raised during the review process.

Please submit your revised manuscript by May 15 2026 11:59PM. If you will need more time than this to complete your revisions, please reply to this message or contact the journal office at digitalhealth@plos.org. When you're ready to submit your revision, log on to https://www.editorialmanager.com/pdig/ and select the 'Submissions Needing Revision' folder to locate your manuscript file.

Please include the following items when submitting your revised manuscript:

* A letter that responds to each point raised by the editor and reviewer(s). You should upload this letter as a separate file labeled 'Response to Reviewers'. This file does not need to include responses to any formatting updates and technical items listed in the 'Journal Requirements' section below.

* A marked-up copy of your manuscript that highlights changes made to the original version. You should upload this as a separate file labeled 'Revised Manuscript with Track Changes'.

* An unmarked version of your revised paper without tracked changes. You should upload this as a separate file labeled 'Manuscript'.

If you would like to make changes to your financial disclosure, competing interests statement, or data availability statement, please make these updates within the submission form at the time of resubmission. Guidelines for resubmitting your figure files are available below the reviewer comments at the end of this letter.

We look forward to receiving your revised manuscript.

Kind regards,

Girum Tareke Zewude, PhD

Academic Editor

PLOS Digital Health

Leo Anthony Celi

Editor-in-Chief

PLOS Digital Health

orcid.org/0000-0001-6712-6626

**Journal Requirements:**

1. As required by our policy on Data Availability, please ensure your manuscript or supplementary information includes the following:

A numbered table of all studies identified in the literature search, including those that were excluded from the analyses.

For every excluded study, the table should list the reason(s) for exclusion.

If any of the included studies are unpublished, include a link (URL) to the primary source or detailed information about how the content can be accessed.

A table of all data extracted from the primary research sources for the systematic review and/or meta-analysis. The table must include the following information for each study:

Name of data extractors and date of data extraction

Confirmation that the study was eligible to be included in the review.

All data extracted from each study for the reported systematic review and/or meta-analysis that would be needed to replicate your analyses.

If data or supporting information were obtained from another source (e.g. correspondence with the author of the original research article), please provide the source of data and dates on which the data/information were obtained by your research group.

If applicable for your analysis, a table showing the completed risk of bias and quality/certainty assessments for each study or outcome.  Please ensure this is provided for each domain or parameter assessed. For example, if you used the Cochrane risk-of-bias tool for randomized trials, provide answers to each of the signalling questions for each study. If you used GRADE to assess certainty of evidence, provide judgements about each of the quality of evidence factor. This should be provided for each outcome.

An explanation of how missing data were handled.

This information can be included in the main text, supplementary information, or relevant data repository. Please note that providing these underlying data is a requirement for publication in this journal, and if these data are not provided your manuscript might be rejected.

2. Please update your online Competing Interests statement. If you have no competing interests to declare, please state: "The authors have declared that no competing interests exist."

3. Please provide separate main figure files in .tif or .eps format only and ensure that all files are under our size limit of 10MB.

For more information about how to convert your figure files please see our guidelines: https://journals.plos.org/digitalhealth/s/figures

4. We have noticed that you have uploaded Supporting Information files, but you have not included a list of legends. Please add a full list of legends for your Supporting Information files before or after the references list.

5. If the reviewer comments include a recommendation to cite specific previously published works, please review and evaluate these publications to determine whether they are relevant and should be cited. There is no requirement to cite these works unless the editor has indicated otherwise.

**Additional Editor Comments (if provided):**

[Note: HTML markup is below. Please do not edit.]

**Reviewers' Comments:**

Reviewer's Responses to Questions

**Comments to the Author**

1. Does this manuscript meet PLOS Digital Health’s publication criteria? Is the manuscript technically sound, and do the data support the conclusions? The manuscript must describe methodologically and ethically rigorous research with conclusions that are appropriately drawn based on the data presented.

Reviewer #1: Yes

Reviewer #2: Yes

**********

2. Has the statistical analysis been performed appropriately and rigorously?

Reviewer #1: Yes

Reviewer #2: Yes

**********

3. Have the authors made all data underlying the findings in their manuscript fully available (please refer to the Data Availability Statement at the start of the manuscript PDF file)?

The PLOS Data policy requires authors to make all data underlying the findings described in their manuscript fully available without restriction, with rare exception. The data should be provided as part of the manuscript or its supporting information, or deposited to a public repository. For example, in addition to summary statistics, the data points behind means, medians and variance measures should be available. If there are restrictions on publicly sharing data—e.g. participant privacy or use of data from a third party—those must be specified.

Reviewer #1: Yes

Reviewer #2: Yes

**********

4. Is the manuscript presented in an intelligible fashion and written in standard English?

PLOS Digital Health does not copyedit accepted manuscripts, so the language in submitted articles must be clear, correct, and unambiguous. Any typographical or grammatical errors should be corrected at revision, so please note any specific errors here.

Reviewer #1: Yes

Reviewer #2: Yes

**********

5. Review Comments to the Author

Please use the space provided to explain your answers to the questions above. You may also include additional comments for the author, including concerns about dual publication, research ethics, or publication ethics. (Please upload your review as an attachment if it exceeds 20,000 characters)

Reviewer #1: Starting on page 31, the content becomes repetitive.

A key limitation is that while the authors compare app features, they do not examine user responses, such as number of downloads or user ratings, which could provide additional insight into app adoption and impact.

The authors may also consider exploring differences by country and, if possible, including the United States or North America, where English is also widely used. In addition, factors such as economic status and population age structure may be relevant and worth considering.

Reviewer #2: Reviewer Report – PLOS Digital Health

Manuscript ID: PDIG-D-25-01183

Title: Digital Mental Health Apps for Young People in Europe: A Systematic Cross-Country Analysis

Summary

This manuscript presents a systematic, cross-country descriptive analysis of mental health–related smartphone applications identified through app store searches conducted across seven European countries and languages. The study addresses an important and timely question concerning linguistic accessibility, commercialization, and the visibility of digital mental health resources encountered by young people in real-world app marketplaces.

The methods are clearly reported and appropriate for the descriptive objectives of the study. However, several interpretive statements extend beyond what can be supported by app store–derived data alone, and the manuscript would benefit from clearer justification of scope, improved consistency in reporting results, and stronger articulation of how the findings inform digital health systems, governance, and equity. I therefore recommend major revision.

Major Comments

1. Justification of Country Selection and Scope

Section: Methods

The title frames the analysis as representing “Europe,” yet only seven countries are included. While this selection may be pragmatic or data-driven, the rationale for how these countries collectively represent Europe is not clearly articulated.

Recommendation:

Please explicitly justify the selection of the seven countries (e.g., geographic spread, linguistic diversity, population size, or app store relevance) and clarify whether findings should be interpreted as illustrative rather than representative of Europe as a whole. This clarification is important to avoid overgeneralization.

2. Alignment Between Data Source and Interpretation

Sections: Results / Discussion

Several statements imply provision, verification, or effectiveness of therapeutic content (e.g., CBT-based interventions, AI-driven personalised support, professional involvement), despite the analysis relying solely on app store descriptions.

Examples of wording patterns that require caution include:

• Claims that apps provide CBT or therapy, rather than claiming to do so

• Statements that AI chatbots deliver personalised mental health support, rather than being described as AI-based

• References to clinical or professional involvement without clarification that this is based on developer claims

• Language implying user benefit or quality (e.g., apps support mental health needs)

Recommendation:

Please review the Results and Discussion to ensure consistent, cautious phrasing throughout, clearly indicating that therapeutic approaches, AI functionality, professional input, and intended benefits are marketed or claimed features, not independently verified functionality. This alignment is essential to ensure conclusions remain fully supported by the data source.

3. Framing Within Digital Health Systems

Section: Discussion

While the Discussion accurately summarizes the findings, it remains largely descriptive and focused on individual app characteristics. For a PLOS Digital Health readership, clearer system-level interpretation would strengthen the manuscript.

Recommendation:

Please strengthen the Discussion to clarify how observed patterns—such as English-language dominance, freemium cost models, and app store visibility—inform understanding of digital health governance, equity, regulation, or implementation for youth mental health services, while remaining within the descriptive scope of the data.

4. Definition and Use of the Term “Young People”

Sections: Methods / Results

The manuscript frequently refers to apps targeting “young people,” although many identified apps appear to be rated for broad age groups rather than explicitly designed for adolescents.

In particular, the phrase “young adults and their trusted adults” requires clarification to confirm whether this wording is accurate and how it aligns with the study’s conceptualization of “young people.”

Recommendation:

Please clarify how “young people” is operationalized in this study and reflect on how app store age ratings and target descriptions affect interpretation, including implications for youth appropriateness and safeguarding.

Minor Comments

5. Abbreviations

Please ensure that all abbreviations are introduced at first use. For example, the abbreviation “VOS” appears without prior definition.

6. Consistency and Accuracy in Reporting Results

Several instances would benefit from improved consistency and numerical accuracy:

• When reporting proportions (e.g., “The majority of apps (n = 85/171)…”), please include the corresponding percentage for consistency.

• Avoid repetitive constructions such as “56 (66%, n = 56/85) of the 85…” and streamline reporting while maintaining clarity.

• Ensure percentage calculations are accurate and appropriately rounded (e.g., 4/85 is closer to 5% than 4%).

• Maintain a consistent reporting format throughout (e.g., (%, n)).

7. Terminology and Wording Clarifications

• Please clarify whether “free” refers to free to download or free to download and use, where relevant to accessibility.

• In the Discussion, please clarify whether the phrase “investigate regular updates” refers to investigating update frequency or to instituting regular updates.

Reference List

The reference list is largely compliant with PLOS Digital Health submission guidelines. Minor consistency checks are recommended, including uniform formatting of organizational authors, confirmation of active URLs, and clear labeling of any preprints or non–peer-reviewed sources.

Recommendation

Major Revision

This recommendation reflects the need for clearer justification of scope, closer alignment between app store–derived data and interpretive claims, and improved consistency and precision in reporting. These revisions are necessary to ensure accurate interpretation and appropriate framing for a PLOS Digital Health readership, rather than indicating fundamental methodological concerns.

**********

6. PLOS authors have the option to publish the peer review history of their article (what does this mean?). If published, this will include your full peer review and any attached files.

**Do you want your identity to be public for this peer review?**  If you choose “no”, your identity will remain anonymous but your review may still be made public.

For information about this choice, including consent withdrawal, please see our Privacy Policy.

Reviewer #1: No

Reviewer #2: No

**********

[NOTE: If reviewer comments were submitted as an attachment file, they will be attached to this email and accessible via the submission site. Please log into your account, locate the manuscript record, and check for the action link "View Attachments". If this link does not appear, there are no attachment files.]

**Figure resubmission:**

While revising your submission, we strongly recommend that you use PLOS’s NAAS tool (https://ngplosjournals.pagemajik.ai/artanalysis) to test your figure files. NAAS can convert your figure files to the TIFF file type and meet basic requirements (such as print size, resolution), or provide you with a report on issues that do not meet our requirements and that NAAS cannot fix.

After uploading your figures to PLOS’s NAAS tool - https://ngplosjournals.pagemajik.ai/artanalysis, NAAS will process the files provided and display the results in the "Uploaded Files" section of the page as the processing is complete. If the uploaded figures meet our requirements (or NAAS is able to fix the files to meet our requirements), the figure will be marked as "fixed" above. If NAAS is unable to fix the files, a red "failed" label will appear above. When NAAS has confirmed that the figure files meet our requirements, please download the file via the download option, and include these NAAS processed figure files when submitting your revised manuscript.

**Reproducibility:**

To enhance the reproducibility of your results, we recommend that authors of applicable studies deposit laboratory protocols in protocols.io, where a protocol can be assigned its own identifier (DOI) such that it can be cited independently in the future. Additionally, PLOS ONE offers an option to publish peer-reviewed clinical study protocols. Read more information on sharing protocols at https://plos.org/protocols?utm_medium=editorial-email&utm_source=authorletters&utm_campaign=protocols

# Author comment: Author response to Decision Letter 1

**DOI:** 10.1371/journal.pdig.0001622.r002

31 Mar 2026

Attachment Submitted filename: Response to Reviewers.pdf

# Peer review: Decision Letter 1

**DOI:** 10.1371/journal.pdig.0001622.r003

**Authors:** Mengyu Wang, Girum Zewude

13 May 2026

PDIG-D-25-01183R1

Digital Mental Health Apps for Young People in Europe: A Systematic Cross-Country Analysis

PLOS Digital Health

Dear Dr. Madrid Cagigal,

Thank you for submitting your manuscript to PLOS Digital Health. After careful consideration, we feel that it has merit but does not fully meet PLOS Digital Health's publication criteria as it currently stands. Therefore, we invite you to submit a revised version of the manuscript that addresses the points raised during the review process.

Please submit your revised manuscript by Jun 12 2026 11:59PM. If you will need more time than this to complete your revisions, please reply to this message or contact the journal office at digitalhealth@plos.org. When you're ready to submit your revision, log on to https://www.editorialmanager.com/pdig/ and select the 'Submissions Needing Revision' folder to locate your manuscript file.

Please include the following items when submitting your revised manuscript:

* A letter that responds to each point raised by the editor and reviewer(s). You should upload this letter as a separate file labeled 'Response to Reviewers'. This file does not need to include responses to any formatting updates and technical items listed in the 'Journal Requirements' section below.

* A marked-up copy of your manuscript that highlights changes made to the original version. You should upload this as a separate file labeled 'Revised Manuscript with Track Changes'.

* An unmarked version of your revised paper without tracked changes. You should upload this as a separate file labeled 'Manuscript'.

If you would like to make changes to your financial disclosure, competing interests statement, or data availability statement, please make these updates within the submission form at the time of resubmission. Guidelines for resubmitting your figure files are available below the reviewer comments at the end of this letter.

As the corresponding author, your ORCID iD is verified in the submission system and will appear in the published article. PLOS supports the use of ORCID, and we encourage all coauthors to register for an ORCID iD and use it as well. Please encourage your coauthors to verify their ORCID iD within the submission system before final acceptance, as unverified ORCID iDs will not appear in the published article. Only the individual author can complete the verification step; PLOS staff cannot verify ORCID iDs on behalf of authors.

We look forward to receiving your revised manuscript.

Kind regards,

Girum Tareke Zewude

Academic Editor

PLOS Digital Health

Mengyu Wang

Section Editor

PLOS Digital Health

Leo Anthony Celi

Editor-in-Chief

PLOS Digital Health

orcid.org/0000-0001-6712-6626

**Journal Requirements:**

1. Please upload separate figure files in .tif or .eps format. Also, remove the figures from your manuscript file but keep the legends.

For more information about figure files please see our guidelines:

https://journals.plos.org/digitalhealth/s/figures

https://journals.plos.org/digitalhealth/s/figures#loc-file-requirements

2. We have noticed that you have uploaded Supporting Information files, but you have not included a list of legends. Please add a full list of legends for your Supporting Information files after the references list.

If the reviewer comments include a recommendation to cite specific previously published works, please review and evaluate these publications to determine whether they are relevant and should be cited. There is no requirement to cite these works unless the editor has indicated otherwise.

**Additional Editor Comments (if provided):**

[Note: HTML markup is below. Please do not edit.]

**Reviewers' Comments:**

Reviewer's Responses to Questions

**Comments to the Author**

1. If the authors have adequately addressed your comments raised in a previous round of review and you feel that this manuscript is now acceptable for publication, you may indicate that here to bypass the “Comments to the Author” section, enter your conflict of interest statement in the “Confidential to Editor” section, and submit your "Accept" recommendation.

Reviewer #2: All comments have been addressed

Reviewer #3: (No Response)

**********

2. Does this manuscript meet PLOS Digital Health’s publication criteria? Is the manuscript technically sound, and do the data support the conclusions? The manuscript must describe methodologically and ethically rigorous research with conclusions that are appropriately drawn based on the data presented.

Reviewer #2: Yes

Reviewer #3: Yes

**********

3. Has the statistical analysis been performed appropriately and rigorously?

Reviewer #2: Yes

Reviewer #3: N/A

**********

4. Have the authors made all data underlying the findings in their manuscript fully available (please refer to the Data Availability Statement at the start of the manuscript PDF file)?

The PLOS Data policy requires authors to make all data underlying the findings described in their manuscript fully available without restriction, with rare exception. The data should be provided as part of the manuscript or its supporting information, or deposited to a public repository. For example, in addition to summary statistics, the data points behind means, medians and variance measures should be available. If there are restrictions on publicly sharing data—e.g. participant privacy or use of data from a third party—those must be specified.

Reviewer #2: Yes

Reviewer #3: Yes

**********

5. Is the manuscript presented in an intelligible fashion and written in standard English?

PLOS Digital Health does not copyedit accepted manuscripts, so the language in submitted articles must be clear, correct, and unambiguous. Any typographical or grammatical errors should be corrected at revision, so please note any specific errors here.

Reviewer #2: Yes

Reviewer #3: Yes

**********

6. Review Comments to the Author

Please use the space provided to explain your answers to the questions above. You may also include additional comments for the author, including concerns about dual publication, research ethics, or publication ethics. (Please upload your review as an attachment if it exceeds 20,000 characters)

**Reviewer #2:**  Please report the results in the following format:

Format:

n/N (%) OR % (n/N)

Example:

312/699 (44.6%) of participants had viral suppression

or

Viral suppression was observed in 312/699 participants (44.6%)

There was an error in the interpretation of my comment. There is still some repetition e.g. Regarding language availability, nine (41%, n=9/22) apps were offered only in English (NOTE nine appears twice. THIS IS BEST PRESENTED AS 41% (9/22); one (5%, n=1/22) - THIS CAN BE PRESENTED AS 5%(1/22). Hope this is clear. YOU CAN CONFIRM WITH PLOS DH FORMATTING GUIDELINES OR PREVIOUS PUBLISHED ARTICLES.

ALL THE BEST

**Reviewer #3:**  The authors, as part of a European research project, examined the availability of mental health apps in their respective countries, focusing on the target group of “young users.”

I was not involved in the review of the first version of the manuscript. However, I have read the previous review reports and the authors’ response, and I have contributed to assessing whether the feedback was adequately addressed. I appreciate the extended discussion, but I would also have liked that the results were presented in a different form, to better prepare for the discussion and aligning better with the research aim.

I recommend looking into the following aspects of the paper, which in my opinion have not been tackled satisfactorily:

1. Target group “young people”

The authors state that the study focuses on young people (from children to adolescents).

The authors indicate that their analysis is based solely on information in the App Store/Play Store: We acknowledge that app store age ratings (4+, 12+, 18+) reflect platform content guidelines rather than intended target populations, and that these do not map straightforwardly onto our conceptualisation of young people (...) . Therefore, age-related classifications reported in this study reflect app store metadata rather than verified target populations.

In other words, the way the app actually looks and functions has not been evaluated. I therefore do not find it convincing when the authors claim to have assessed apps specifically for young people with mental health problems. I would rather distinguish between apps that do not specify a clear target group and apps that do; one of the results could then be the number of apps that explicitly target young people, this would make the “target population” column in Table 1 (appendix) and table 3 (manuscript) more meaningful. An age rating such as “4+” does not necessarily mean that an app is intended for use by children/teenagers or has been developed with this group in mind. Are the language, look and feel, and the type of interventions tailored to this target group? Probably not. However, I consider the research team sufficiently qualified to also make a substantive judgement about the suitability of these “4+apps” for young people.

2. Tables, visualisations, and cross-country differences

The tables in the appendix are currently purely descriptive. I recommend using the tables more actively in the Results section and, for example, adding other types of visualisation and colour coding to illustrate differences between what an English-language search versus a native-language search yields in terms of apps. For example, could the contrast between apps in English and in the local language be developed further? For example, by explicitly comparing availability, types of interventions, or target groups per language category.

One of the aims was to identify cross-country differences. Because the data are not presented by country in the Results, I feel that these cross-country differences have not yet been sufficiently elaborated, although the Discussion points at some interesting findings. For example, the authors state:

This pattern underscores the global dominance of English-language apps and suggests limited localisation or cultural adaptation.

This conclusion would be stronger if cross-country patterns were more clearly shown in the results, e.g. in a graph or other visual.

Table 2 (Manuscript)

The main point the authors seek to make with Table 2 in the Results section is not clear to me in terms of the article’s central message, because it is about one specific app.

Table 3 (manuscript):

• I would reconsider the age-based categorisation, for the reasons outlined under point 1 above.

• Is it relevant for this article that some international apps are also available in Japanese, Arabic, etc.? It seems to me that three categories would be sufficient: “English only,” “local language only” (from the countries in this study), and “several languages.”

• Is it possible to retrieve information about where the apps originate from—that is, in which country they were developed? This would add a useful dimension to the analysis.

• Is it not somewhat surprising that apps providing access to therapy are free of charge, given that this appears to be the most resource-intensive form of care? Presumably, users have to pay for the consultation itself, or the app is only available to people who already have a care contract. This is an important nuance to highlight when discussing this table and in the Discussion.

All in all I feel the paper has improved, but the results section is too descriptive.

**********

7. PLOS authors have the option to publish the peer review history of their article (what does this mean?). If published, this will include your full peer review and any attached files.

**Do you want your identity to be public for this peer review?**  If you choose “no”, your identity will remain anonymous but your review may still be made public.

For information about this choice, including consent withdrawal, please see our Privacy Policy.

Reviewer #2: **Yes:** ERIC MUGAMBI NTURIBI

Reviewer #3: No

**********

[NOTE: If reviewer comments were submitted as an attachment file, they will be attached to this email and accessible via the submission site. Please log into your account, locate the manuscript record, and check for the action link "View Attachments". If this link does not appear, there are no attachment files.]

**Figure resubmission:**

While revising your submission, we strongly recommend that you use PLOS’s NAAS tool (https://ngplosjournals.pagemajik.ai/artanalysis) to test your figure files. NAAS can convert your figure files to the TIFF file type and meet basic requirements (such as print size, resolution), or provide you with a report on issues that do not meet our requirements and that NAAS cannot fix.

After uploading your figures to PLOS’s NAAS tool - https://ngplosjournals.pagemajik.ai/artanalysis, NAAS will process the files provided and display the results in the "Uploaded Files" section of the page as the processing is complete. If the uploaded figures meet our requirements (or NAAS is able to fix the files to meet our requirements), the figure will be marked as "fixed" above. If NAAS is unable to fix the files, a red "failed" label will appear above. When NAAS has confirmed that the figure files meet our requirements, please download the file via the download option, and include these NAAS processed figure files when submitting your revised manuscript.

**Reproducibility:**

To enhance the reproducibility of your results, we recommend that authors of applicable studies deposit laboratory protocols in protocols.io, where a protocol can be assigned its own identifier (DOI) such that it can be cited independently in the future. Additionally, PLOS ONE offers an option to publish peer-reviewed clinical study protocols. Read more information on sharing protocols at https://plos.org/protocols?utm_medium=editorial-email&utm_source=authorletters&utm_campaign=protocols

# Author comment: Author response to Decision Letter 2

**DOI:** 10.1371/journal.pdig.0001622.r004

22 May 2026

Attachment Submitted filename: Round 2. Response to Reviewers (3).docx

# Peer review: Decision Letter 2

**DOI:** 10.1371/journal.pdig.0001622.r005

**Authors:** Mengyu Wang, Girum Zewude

14 Jul 2026

Digital Mental Health Apps for Young People in Europe: A Systematic Cross-Country Analysis

PDIG-D-25-01183R2

Dear Miss Madrid Cagigal,

We are pleased to inform you that your manuscript 'Digital Mental Health Apps for Young People in Europe: A Systematic Cross-Country Analysis' has been provisionally accepted for publication in PLOS Digital Health.

Before your manuscript can be formally accepted you will need to complete some formatting changes, which you will receive in a follow-up email from a member of our team.

Please note that your manuscript will not be scheduled for publication until you have made the required changes, so a swift response is appreciated.

IMPORTANT: The editorial review process is now complete. PLOS will only permit corrections to spelling, formatting or significant scientific errors from this point onwards. Requests for major changes, or any which affect the scientific understanding of your work, will cause delays to the publication date of your manuscript.

If your institution or institutions have a press office, please notify them about your upcoming paper to help maximize its impact. If they'll be preparing press materials, please inform our press team as soon as possible -- no later than 48 hours after receiving the formal acceptance. Your manuscript will remain under strict press embargo until 2 pm Eastern Time on the date of publication. For more information, please contact digitalhealth@plos.org.

Thank you again for supporting Open Access publishing; we are looking forward to publishing your work in PLOS Digital Health.

Best regards,

Girum Tareke Zewude, PhD

Academic Editor

PLOS Digital Health

***********************************************************

**Additional Editor Comments (if provided):**

**Reviewer Comments (if any, and for reference):**

Reviewer's Responses to Questions

**Comments to the Author**

1. If the authors have adequately addressed your comments raised in a previous round of review and you feel that this manuscript is now acceptable for publication, you may indicate that here to bypass the “Comments to the Author” section, enter your conflict of interest statement in the “Confidential to Editor” section, and submit your "Accept" recommendation.

Reviewer #3: All comments have been addressed

Reviewer #4: All comments have been addressed

Reviewer #5: All comments have been addressed

**********

2. Does this manuscript meet PLOS Digital Health’s publication criteria? Is the manuscript technically sound, and do the data support the conclusions? The manuscript must describe methodologically and ethically rigorous research with conclusions that are appropriately drawn based on the data presented.

Reviewer #3: Yes

Reviewer #4: Yes

Reviewer #5: Yes

**********

3. Has the statistical analysis been performed appropriately and rigorously?

Reviewer #3: N/A

Reviewer #4: Yes

Reviewer #5: Yes

**********

4. Have the authors made all data underlying the findings in their manuscript fully available (please refer to the Data Availability Statement at the start of the manuscript PDF file)?

The PLOS Data policy requires authors to make all data underlying the findings described in their manuscript fully available without restriction, with rare exception. The data should be provided as part of the manuscript or its supporting information, or deposited to a public repository. For example, in addition to summary statistics, the data points behind means, medians and variance measures should be available. If there are restrictions on publicly sharing data—e.g. participant privacy or use of data from a third party—those must be specified.

Reviewer #3: Yes

Reviewer #4: Yes

Reviewer #5: Yes

**********

5. Is the manuscript presented in an intelligible fashion and written in standard English?

PLOS Digital Health does not copyedit accepted manuscripts, so the language in submitted articles must be clear, correct, and unambiguous. Any typographical or grammatical errors should be corrected at revision, so please note any specific errors here.

Reviewer #3: Yes

Reviewer #4: Yes

Reviewer #5: Yes

**********

6. Review Comments to the Author

Please use the space provided to explain your answers to the questions above. You may also include additional comments for the author, including concerns about dual publication, research ethics, or publication ethics. (Please upload your review as an attachment if it exceeds 20,000 characters)

Reviewer #3: All my comments and concerns have been adequately addressed; the paper has more analytical depth now. Good luck with further research into this topic, I hope your empirical research project will lead to interesting results

Reviewer #4: The authors have responded comprehensively to the reviewers' comments and have incorporated the major requested revisions into the manuscript. The revisions substantially strengthen the paper, particularly with respect to clarification of scope, interpretation of app-store-derived data, the conceptualisation of young people, and the presentation of cross-country and cross-language findings. While some suggestions were only partially implemented (e.g., additional visualisation of cross-country differences), the principal concerns raised during review have been addressed satisfactorily.

Reviewer #5: Great paper! I wonder if you were thinking about expanding more on policies of the apps related to data privacy and usage, especially when they had AI enabled chatbots.

**********

7. PLOS authors have the option to publish the peer review history of their article (what does this mean?). If published, this will include your full peer review and any attached files.

**Do you want your identity to be public for this peer review?**  If you choose “no”, your identity will remain anonymous but your review may still be made public.

For information about this choice, including consent withdrawal, please see our Privacy Policy.

Reviewer #3: No

Reviewer #4: **Yes:** Parvati Naliyatthaliyazchayil

Reviewer #5: **Yes:** Salman Khan

**********
